# From Waste to Wealth: A Review of Eutectic Molten Salt Method for Direct Regeneration of Spent Lithium‐Ion Battery

**DOI:** 10.1002/advs.202504609

**Published:** 2025-06-05

**Authors:** Junyi Wang, Zehua Qi, Qi Zhao, Kaimin Shih

**Affiliations:** ^1^ Department of Civil Engineering The University of Hong Kong Hong Kong Hong Kong SAR China

**Keywords:** cathode, direct regeneration, eutectic molten salt method, lithium‐ion batteries

## Abstract

The rapid growth of electric vehicles (EVs) has significantly increased the demand for lithium (Li)‐ion batteries (LIBs), bringing environmental, economic, and technical challenges. Developing recycling methods that ensure economic viability and reduce environmental impact is now critical. Traditional hydrometallurgical and pyrometallurgical routes, while established and widely applied for LIB recycling, generate substantial volumes of waste (wastewater, slag, toxic gas) and low‐value chemical components, e.g., Li_2_CO_3_, Nickel (Ni)/Cobalt (Co)/Manganese (Mn) salts, limiting their sustainability. Nowadays, the eutectic molten salt method, a typical direct regeneration technology, is gaining attention, standing out for its non‐destructive repair, cost‐effectiveness, and environmental benefits. It allows flexible salt combinations, adjustable lithiation, and annealing temperatures, and the use of additives to meet specific recycling needs, improving the electrochemical performance of spent cathode materials. This review begins with an overview of LIB composition and degradation mechanisms, then delves into recent advances in the eutectic molten salt method, covering pre‐treatment, salt selection, thermal optimization, and cost‐benefit analysis. In addition, these eutectic molten salt methods are compared with traditional hydrometallurgical and pyrometallurgical methods in terms of both economic and environmental impacts. Finally, the considerable industrial potential of eutectic molten salt methods for LIB recycling is highlighted, especially today when the EV sector is booming.

## Graphical abstract

The main process of the eutectic molten salt method and the direct regeneration of Spent lithium (Li)‐ion batteries by the eutectic molten salt method have brought structural, performance, environmental, and economic benefits.

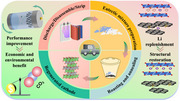

## 1 Introduction

**Figure 1:**
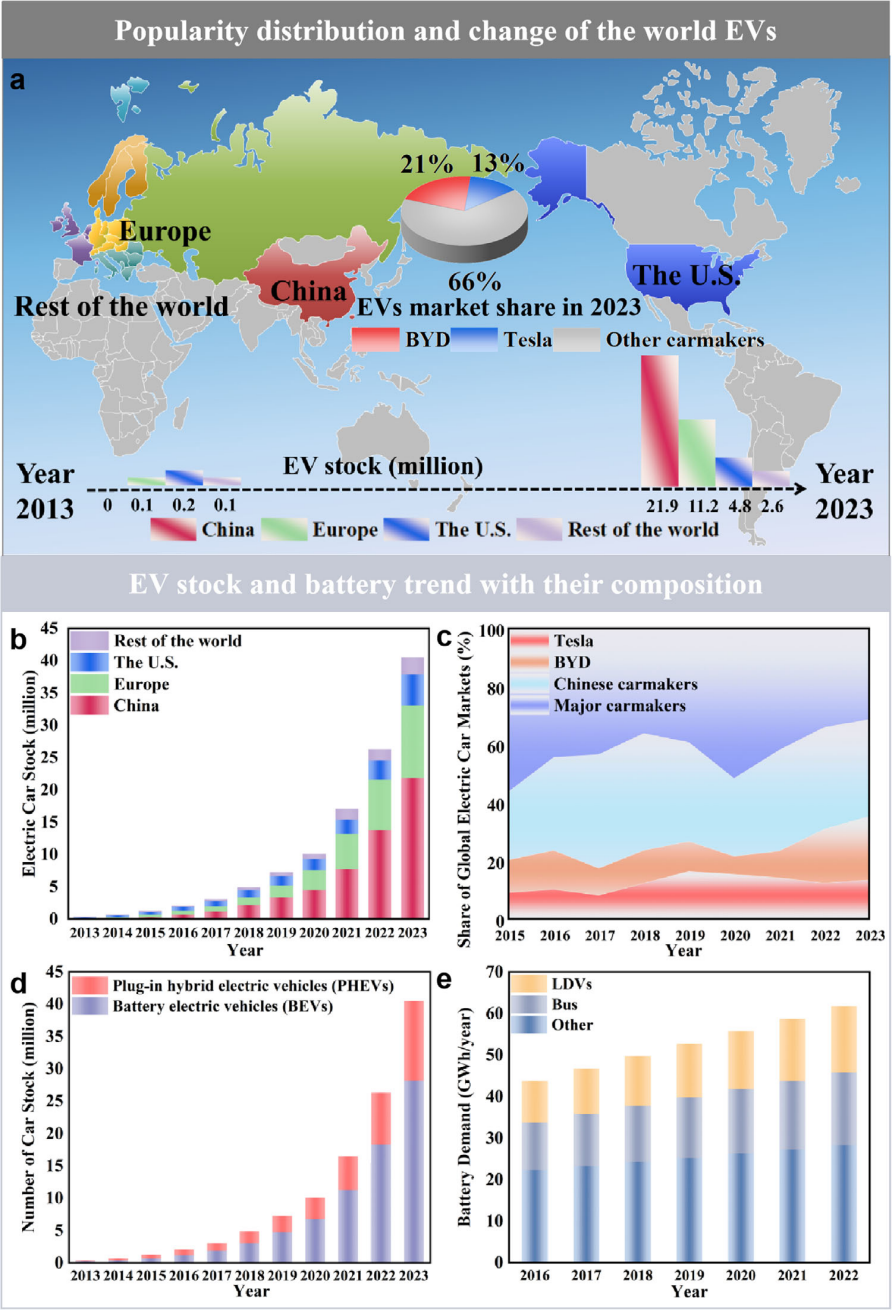
EV sales from 2013 to 2023 and the trend of battery demand. Data source: Global EV Outlook 2023 and 2024. a) Overview of the popularity distribution and change of the world EVs. b) Market share of the world EV stock, illustrated by the country or region. c) Share of global EV markets by selected carmakers. d) Market share of the world EV stock, illustrated by the type of EVs. e) The world battery trends.

Growing environmental and energy issues, as well as the inherent drawbacks of traditional means of transportation in these aspects accelerate the development of EVs.^[^ ^1^ ^]^ The advantages of EVs in durability, cost‐effectiveness, power performance, safety, and energy density, combined with their potential to significantly reduce greenhouse gas emissions, have led to significantly increasing market share (**Figure** **1**). As the global EV stock is projected to reach between 40 and 70 million by 2025,^[^ ^2^ ^]^ the significance of addressing the challenge of spent battery disposal becomes evident. While lithium (Li)‐ion batteries provide significant support for the operation of electric vehicles, the skyrocketing number of electric vehicles has also led to the proliferation of spent Li‐ion batteries.^[^ ^3^ ^]^

**Figure 2:**
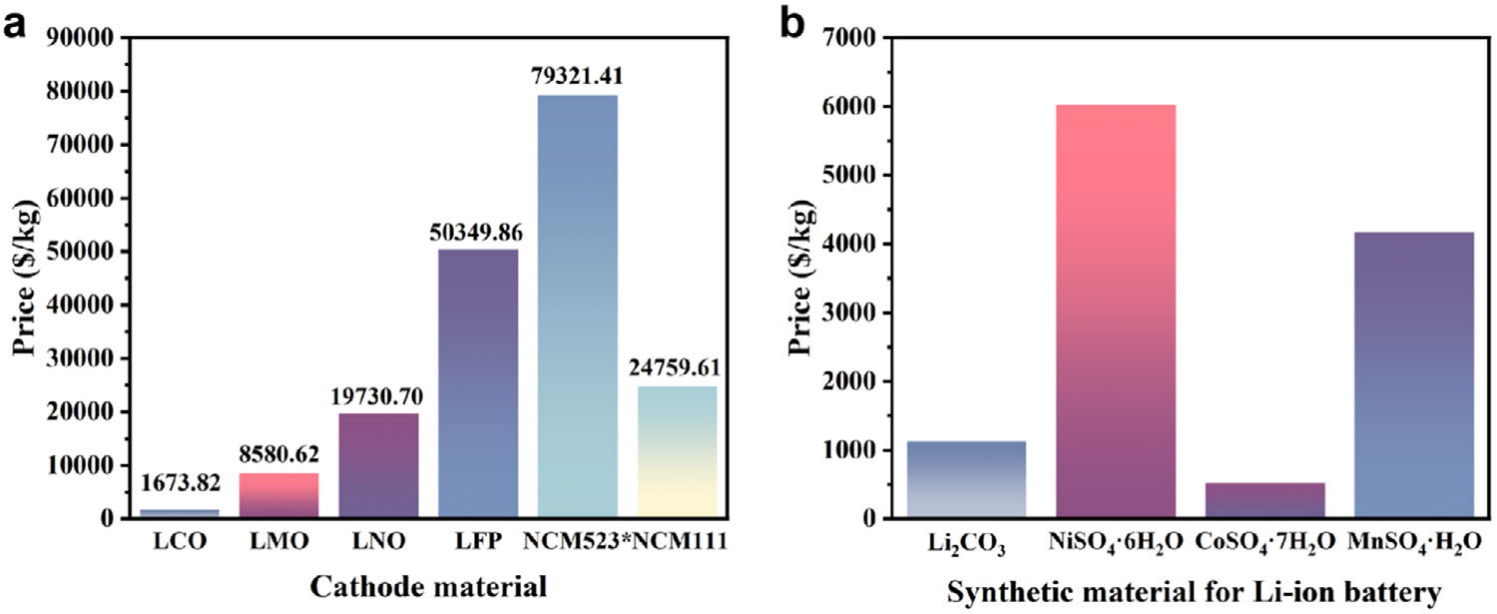
Overview of battery cathode materials and related metal costs. a) The retail price of common lithium battery cathode materials, which refers to Sigma‐Aldrich. And LCO is lithium cobalt oxides (LiCoO_2_), LMO is lithium manganate (LiMn_2_O_4_), LNO is Lithium nickelate (LiNiO_2_), LFP is lithium iron (Fe) phosphate (LiFePO_4_), NCM523 and NCM111 are ternary material LiNi_0.33_Co_0.33_Mn_0.33_O_2_ and ternary material LiNi_0.5_Co_0.2_Mn_0.3_O_2_. b) The price of materials commonly used to synthesize Li‐ion batteries, which refers to Sigma‐Aldrich. Note: If the material is not sold by weight, a normalization calculation is made based on its size and the amount of material per unit area.

Accompanying forecasts indicate that the global volume of spent LIBs is expected to escalate from 10 700 tons in 2012 to a staggering 464 000 tons by 2025.^[^ ^4^ ^]^ Worryingly, <6% of spent LIBs are currently recycled worldwide, with most ending up in landfills.^[^ ^5^ ^]^ The environmental ramifications are significant, as the presence of toxic heavy metals such as cobalt (Co) and nickel (Ni) within batteries poses a substantial risk of contamination to both soil and aquatic systems.^[^ ^6^ ^]^ The residual electrical charge within these spent batteries constitutes a safety concern, with the potential to precipitate explosions or incendiary events.^[^ ^7^ ^]^ Furthermore, these discarded LIBs contain valuable critical elements as secondary resources, with concentrations exceeding those found in natural resources by an order of magnitude greater than ten. Economic rationale is equally compelling, as the recycling of cathode materials from waste batteries is significantly more cost‐effective than the procurement of virgin resources.^[^ ^8^ ^]^ Common cathode materials are evidently valuable. It is not difficult to see from **Figure** **2**a that NCM and LFP are more expensive, of which NCM523 is as high as $79321.41 kg^−1^. At the same time, the salts used to synthesize these cathode materials are also expensive with the Ni salt reaching a costly price (Figure 2b). To mitigate these environmental pressures and the strain on metal resources, an increasing number of countries are placing greater emphasis on the recycling and utilization of Li‐ion batteries and have established relevant regulations. For instance, the European Union mandates the recycling of 45% of rechargeable batteries, with the stipulation that half of these must be recycled and repurposed.^[^ ^9^ ^]^ China is restricting battery disposal in landfills.^[^ ^10^ ^]^ The US Department of Energy (DOE) has issued two notices of intent to provide funding in 2022 to facilitate the production of advanced batteries, including investments in recycling facilities. This initiative underscores the strategic importance of bolstering the domestic battery manufacturing industry and enhancing the sustainability of battery lifecycle management through recycling efforts in America.^[^ ^11^ ^]^ Moreover, there is a growing demand for Li‐ion batteries, and from the perspective of environmental and resource sustainability, the recycling of waste Li‐ion batteries is becoming increasingly imperative.

Conventional pyrometallurgical and hydrometallurgical methods are the mainstream battery recycling methods. The pyrometallurgical method is a rather matured craft stemming from metal smelting; yet it is energy‐intensive and relatively low efficient since most of the valuable metal resources are obtained in the form of metal alloy and partial Li is ended up in slags.^[^ ^12^ ^]^ Hydrometallurgical techniques enable the recovery of various metallic elements in higher purities, but at the expense of consuming significant amounts of chemical reagents. This leads to high costs, complex recycling processes, and environmental issues such as water pollution.^[^ ^13^ ^]^ Both approaches still face challenges stemming from technical bottlenecks, economic feasibility, energy consumption, and environmental impact. Additionally, these destructive recycling processes smelt or dissolve the batteries and decompose the cathode materials into low‐value elemental constituents, such as Li_2_CO_3_ and CoCO_3_,^[^ ^14^ ^]^ which lead to high processing cost and economic loss of the product.^[^ ^15^ ^]^

**Figure 3:**
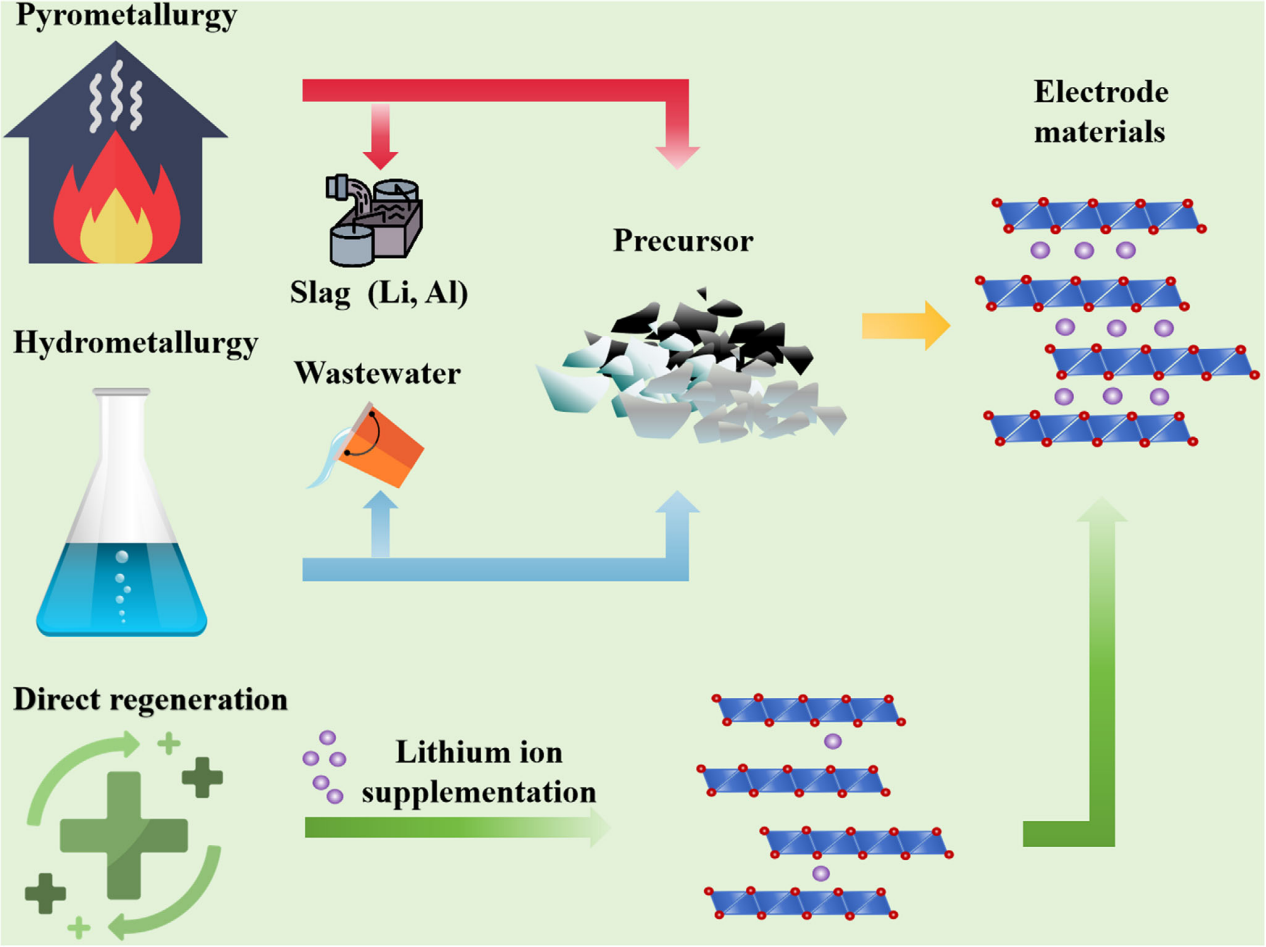
Process schematic of three recycling routes, pyrometallurgy, hydrometallurgy, and direct regeneration.

Recently, a breakthrough came in the form of a direct regeneration strategy that restores the electrochemical performance of degraded cathode material through Li supplementation and crystalline restoration. Direct regeneration technology is a method to heal the composition and structure defects of spent cathode materials by adding a certain number of raw materials into the cathode materials with a post‐treatment, to obtain new cathode materials. Since defects in the electrode material can directly restore its crystal structure without destroying the original crystal structure of the material, lengthy and expensive purification steps can be avoided (as shown in **Figure** **3**).^[^ ^16^ ^]^ The method ensures that valuable metals within the degraded cathode are preserved and can be used directly after regeneration, with a theoretical Li recovery rate approaching 100%.^[^ ^17^ ^]^ Furthermore, compared to conventional recycling methods, this process significantly reduces energy consumption, greenhouse gas emissions, and other environmental impacts.^[^ ^18^ ^]^

In recent years, direct regeneration, offering clear‐cut advantages, has become a key approach for recovering Li‐ion battery cathode materials. Over this period, the eutectic molten salt method has made significant progress, proving highly effective in cutting recycling costs and enhancing product efficiency. Within this review, a comprehensive examination of the eutectic molten salt method's mechanism, benefits, and prospects is presented. Efforts have been made to compile the recent progress and relevant accomplishments in the direct regeneration of Li‐ion battery cathode materials. Furthermore, the performance of refurbished batteries generated through various recycling and regeneration techniques has been analyzed and compared. Additionally, a preliminary quantitative assessment of cost consumption and production efficiency within the battery recycling process has been conducted. The goal is to identify and summarize recycling methods that can harmoniously integrate product quality and performance with environmental sustainability and economic viability. Building on current technological foundations, prospects are discussed to explore the potential for technological improvements. This exploration paves the way for proposing a more streamlined, direct, and energy‐efficient operational model in the future.

## 2 Structure and Composition of Li‐Ion Battery

**Figure 4:**
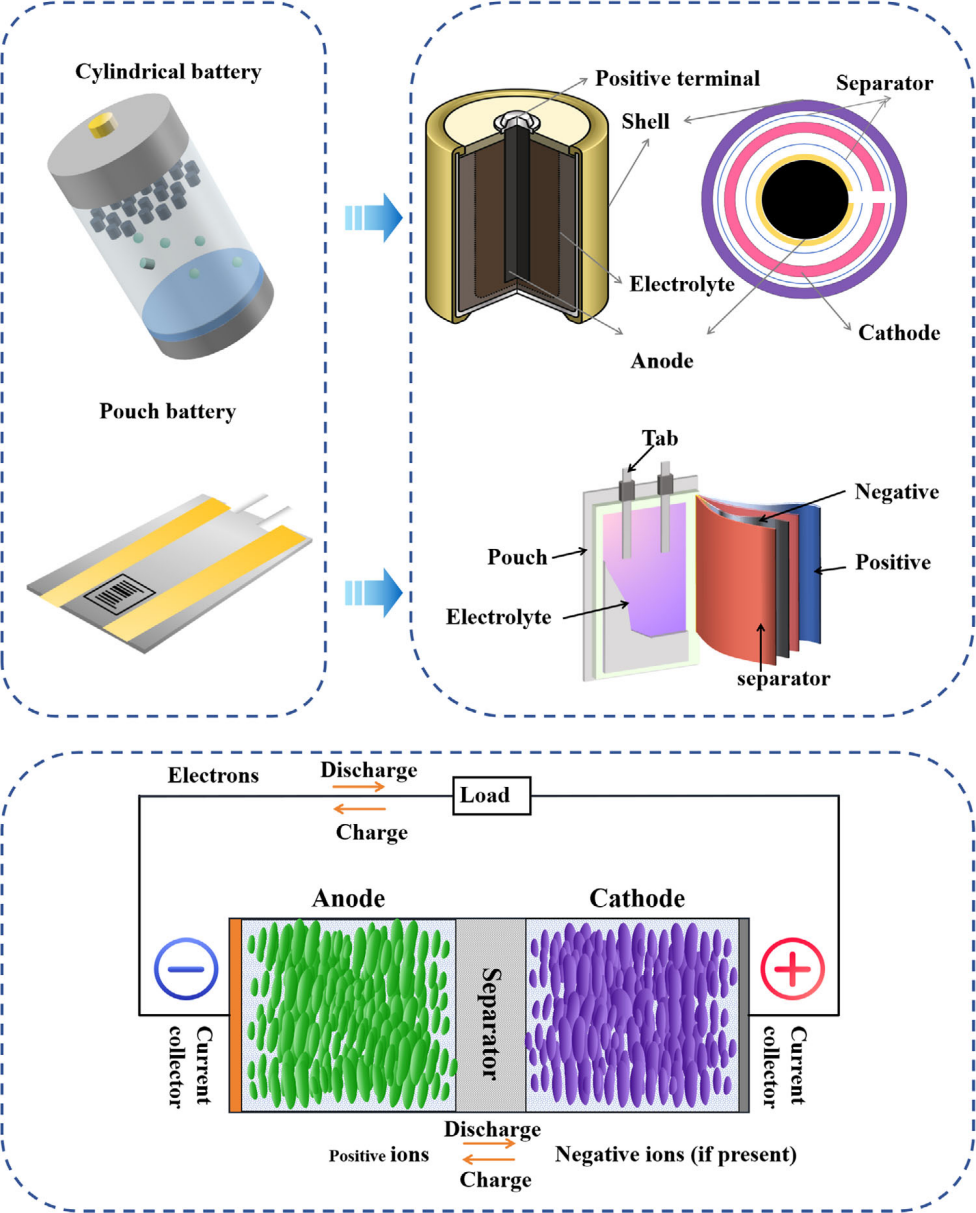
Basic information of battery internal structure and composition and overview of battery internal operating principles.^[^ ^22^ ^]^

Unlike the polymerized Li‐ion batteries used in mobile phones, electric cars are generally equipped with ternary Li batteries with high energy density as power support. These LIBs are a type of electrochemical batteries that release energy through an internal electrochemical reaction.^[^ ^19^ ^]^ Generally, the forms of batteries can include cylindrical battery, pouch battery, button battery and cartridge battery. Taking cylindrical battery and pouch battery as examples, the structural core of the cylindrical battery includes an anode, a cathode, an electrolyte, a separator, a fluid collector, and a battery shell,^[^ ^20^ ^]^ and the pouch battery just wears a pouch instead of shell, which is shown in **Figure** **4**. As the battery works, Li^+^ moves back and forth in the electrolyte: during charging, Li^+^ detaches from the anode and is embedded in the cathode; In a discharge, Li^+^ are released from the cathode and returned to the anode, while electrons are moved from the cathode to the anode through an external circuit, providing power to the external device,^[^ ^20^, ^21^ ^]^ which can also be illustrated by the lower part of Figure 4.

Currently, Li‐ion batteries are common types of lithium batteries among others. (Solid‐state Li batteries, lithium–sulfur (Li–S) batteries, and Li‐air batteries have also garnered widespread attention, but this study does not delve into a discussion of them.) LIBs typically utilize graphite as the anode material, while the cathode materials can consist of LiCoO_2_, LiMn_2_O_4_, LiNiO_2_, nickel‐manganese (Mn)‐cobalt oxide (LiNi_x_Mn_y_Co_z_O_2_), or nickel‐cobalt‐aluminum oxide (LiNi_x_Co_y_Al_z_O_2_), and so on.^[^ ^23^ ^]^ Lithium iron phosphate battery is a battery with LiFePO_4_ as the cathode material, which is favored for its excellent thermal stability and cycle life, and is often used in electric vehicles and large energy storage systems.^[^ ^24^ ^]^

### 2.1 Main Compositions of Battery

Cathode materials possess distinct crystal structures, which directly influence their functional properties, advantages, and limitations. These structural variations are intrinsically linked to their degradation mechanisms. To better understand these relationships, a preliminary analysis was conducted, focusing on the interplay between crystal structure, performance characteristics, and degradation behavior. This investigation aims to establish a foundational framework for identifying and selecting appropriate recycling strategies tailored to the specific properties and degradation patterns of different cathode materials.

#### 2.1.1 Cathode and Anode

**Figure 5:**
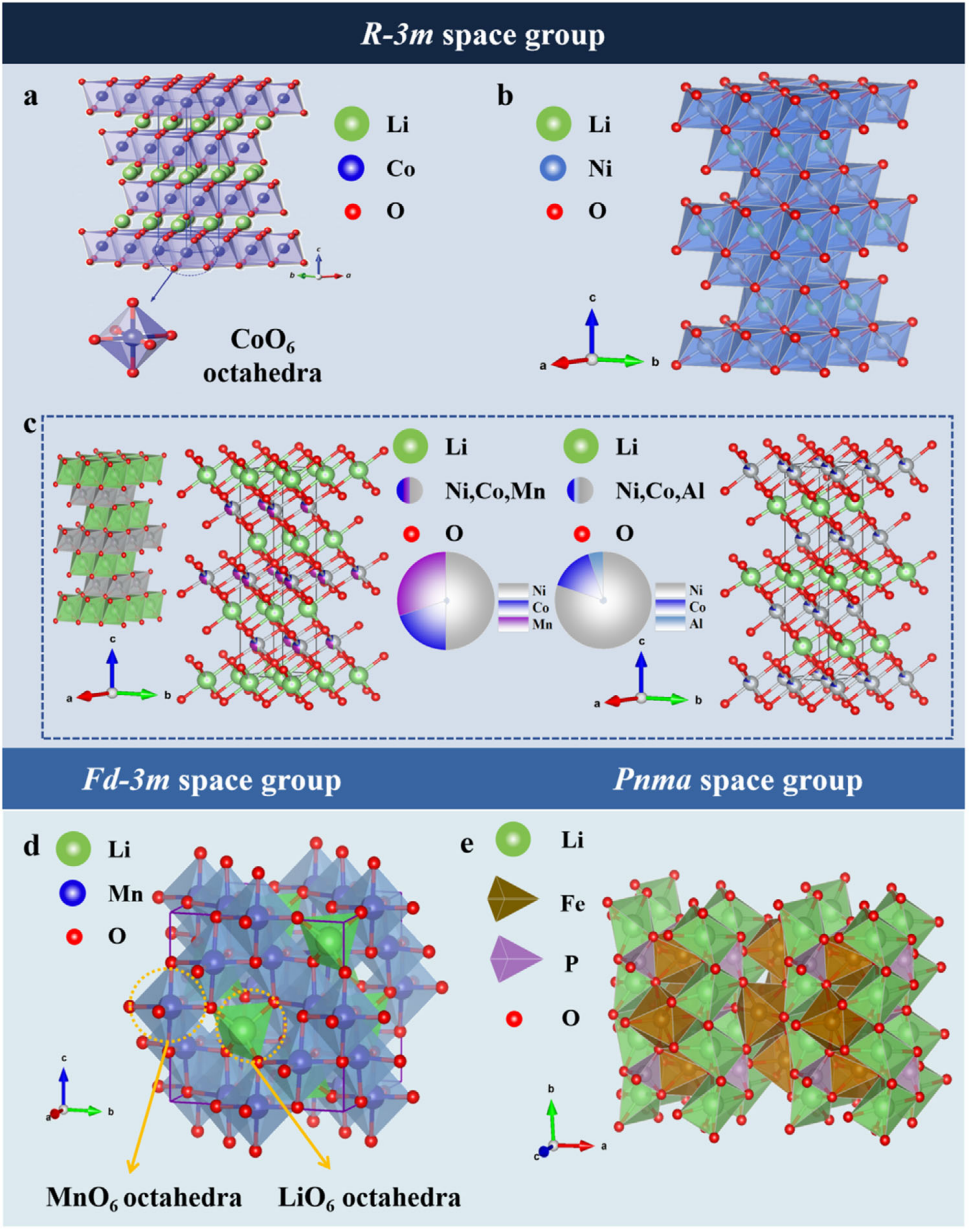
a) Schematic illustration of the crystal structure of LiCoO_2._ Reproduced with permission.^[^ ^25^ ^]^ Copyright 2023, Wiley‐VCH. b) Schematic illustration of the crystal structure of LiNiO_2_ and the graph is drawn based on the data gained from ICSD. c) Schematic illustration of the crystal structure of NCM (LiNi_0.5_Co_0.2_Mn_0.3_O_2_) and NCA (LiNi_0.8_Co_0.15_Al_0.05_O_2_), drawing data are gained from ICSD. d) Schematic illustration of the crystal structure of LiMn_2_O_4_ and the structure on the right is drawn based on the data gained from ICSD. e) Schematic illustration of the crystal structure of LFP, which is drawn based on the data gained from ICSD.

LiCoO_2_ (LCO) (**Figure** **5**a) crystallizes in a layered structure (space group *R‐3m*), where edge‐sharing CoO_6_ octahedra form two‐dimensional sheets with Li⁺ ions migrating between the interlayers. This cathode material exhibits advantages including low production cost and extended cycle life (ranging from hundreds to thousands of cycles).^[^ ^25^ ^]^ LiNiO_2_ (LNO) (Figure 5b) adopts a similar layered structure (space group *R‐3m*) to LCO, but with Ni substituting for Co.^[^ ^26^ ^]^ This substitution enhances the theoretical capacity (∼200 mAh g^−1^ at 4.2 V vs Li⁺/Li) due to the higher redox activity of Ni^3+^/Ni^4+^.^[^ ^27^ ^]^ Lithium nickel‐cobalt manganate (NMC/NCM) (the left of Figure 5c) crystallizes in the *R‐3m* structure with transition metal (Ni/Mn/Co) cations occupying octahedral sites in either random or ordered arrangements within the slabs. These cathode materials deliver high energy densities (200–260 Wh kg^−1^) and exhibit superior rate capability, making them suitable for fast‐charging applications.^[^ ^28^ ^]^ Their compositions are typically classified as NCM111, NCM523, NCM811, etc., based on the stoichiometric ratios of transition metals. Lithium nickel‐cobalt aluminate (NCA) (the right of Figure 5c) adopts an isostructural *R‐3m* layered framework with partial substitution of Mn by Al. This chemical modification enhances thermal stability and energy density compared to conventional NCM systems.^[^ ^29^ ^]^ Conversely, LiMn_2_O_4_ (LMO) has a spinel structure, corresponding to the *Fd‐3m* space group (Figure 5d).^[^ ^30^ ^]^ These batteries are low‐cost, thermally stable, and perform well under extreme conditions.^[^ ^31^ ^]^ LiFePO_4_ (LFP) (Figure 5e) is defined by an olivine structure, which is categorized under the *Pnma* space group, featuring a robust FePO_4_ framework that ensures exceptional cycling stability (2000–5000 cycles).^[^ ^32^ ^]^

For anode, graphite, a prevalent anode material, exhibits a layered structure (P6_3/mmc space group) with sp^2^‐hybridized carbon networks held by van der Waals forces. Its interlayer spacing facilitates reversible Li⁺ intercalation (0.01–0.2 V vs Li⁺/Li), ensuring high capacity and voltage stability.^[^ ^33^ ^]^

#### 2.1.2 Shell, Separator and Electrolyte

Common battery shells include plastic shells, metal shells, aluminum (Al) alloy shells, and the Al‐plastic composite film shells used in pouch cells.^[^ ^21^, ^34^ ^]^ For plastic shells, polyvinyl chloride (PVC) offers cost efficiency, light weight, and effective electrical insulation to prevent short circuits. However, PVC demonstrates limited chemical resistance and impact strength under harsh conditions.^[^ ^35^ ^]^ Metal shells (e.g., stainless steel or iron) provide superior mechanical strength and impact resistance but suffer from heavy weight and suboptimal heat dissipation.^[^ ^36^ ^]^ Al alloy shells combine light weight with mechanical durability, featuring self‐forming anti‐corrosion oxide layers and improved thermal conductivity, though at higher costs.^[^ ^37^ ^]^ Al‐plastic composite shells deliver exceptional light weight and safety through controlled expansion/rupture failure mechanisms rather than explosive failure while enabling greater volumetric capacity than rigid shells‐albeit with similar cost disadvantages.^[^ ^38^ ^]^

The separator physically isolates cathode and anode to prevent short circuits while enabling ion transport through its porous structure. Common materials like polyethylene (PE) and polypropylene (PP) provide chemical/thermal stability, resisting electrolyte corrosion and maintaining integrity at high temperatures to mitigate overheating risks.^[^ ^39^ ^]^

For LIBs, common types of electrolytes include non‐aqueous electrolytes, gel polymer electrolytes, solid‐state electrolytes, and aqueous electrolytes.^[^ ^40^ ^]^ Non‐aqueous electrolytes mainly consist of organic carbonate solvents include ethylene carbonate (EC), dimethyl carbonate (DMC), and propylene carbonate (PC), while frequently used Li salts are lithium hexafluorophosphate (LiPF_6_) and lithium tetrafluoroborate (LiBF_4_), which offer high ionic conductivity, good wettability, and form stable SEI layers, improving battery cycle life and performance.^[^ ^41^ ^]^ Gel polymer electrolytes are composed of a polymer matrix, Li salts, organic solvents, and specific additives. By adjusting the ratio of the polymer matrix to Li salts, the electrolyte can be gelled. Common polymer matrices include polyvinyl alcohol, polyacrylic acid, and so forth.^[^ ^40b^ ^]^ Solid‐state electrolytes are categorized into polymer‐based (e.g., polyethylene oxide with Li salts) and inorganic types (e.g., Li_7_La_3_Zr_2_O_12_ oxide or Li_10_GeP_2_S_12_ sulfide). Polymer electrolytes provide mechanical strength, while inorganic ones offer superior electrochemical stability.^[^ ^40^, ^42^ ^]^ Aqueous electrolytes use water as the solvent with LiTFSI as the primary Li salt, along with additives for overcharge protection and flame retardancy.^[^ ^40b^ ^]^

### 2.2 Degradation Mechanisms of Li‐Ion Battery Components

#### 2.2.1 Degradation of Electrode Materials

The degradation of cathode materials is influenced by a multitude of factors. In addition to extrinsic factors such as corrosion by the electrolyte, thermal degradation due to high temperatures, and performance deterioration caused by overcharging and overdischarging, intrinsic changes within the material's internal structure are also of significant importance.^[^ ^43^ ^]^

**Figure 6:**
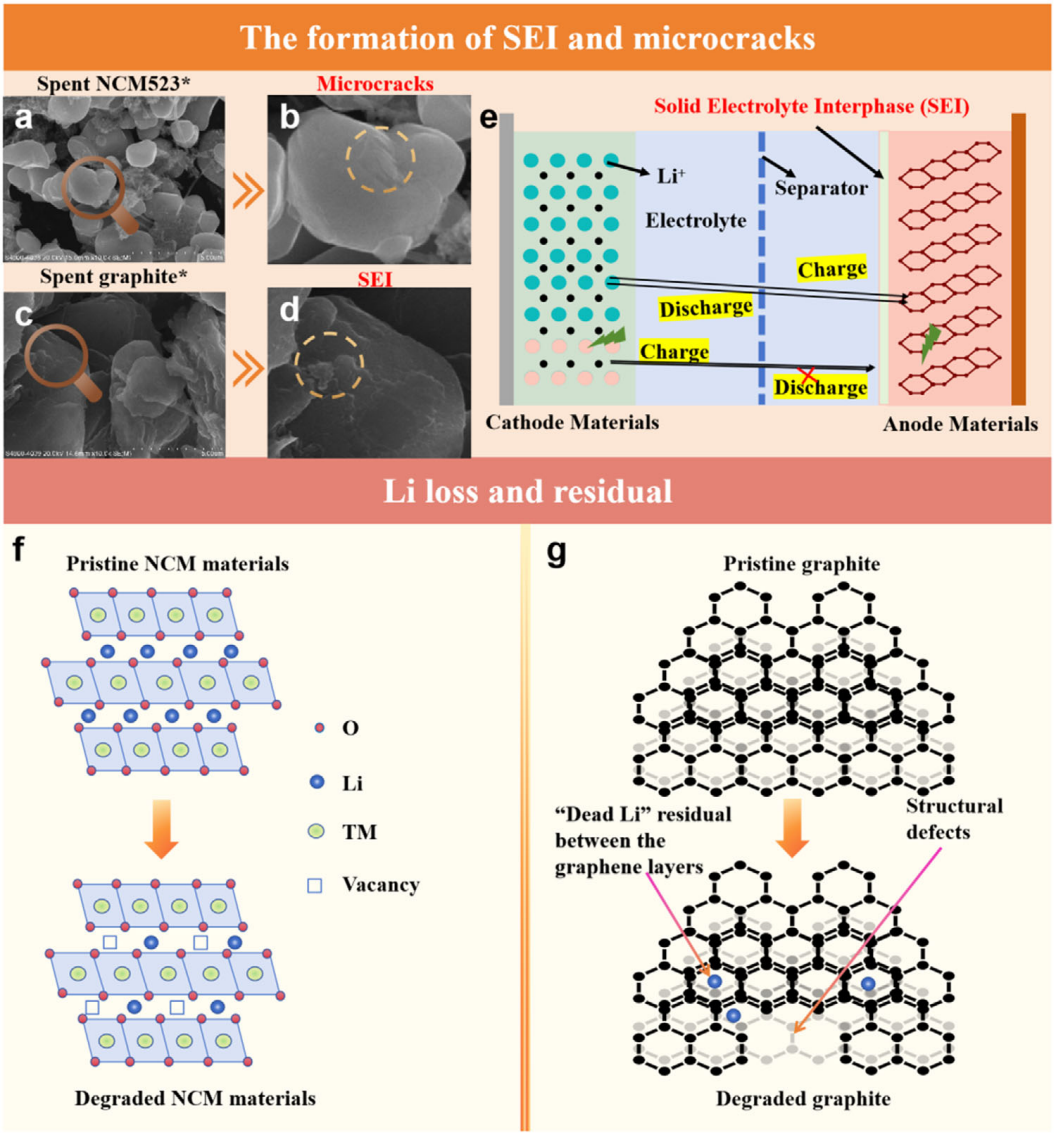
Main degradation mechanisms of NCM cathode material and graphite anode. a) The scanning electron microscope (SEM) image of spent NCM523, and b) the microcrack on it. c) The SEM image of spent graphite, and d) the SEI on it. e) Schematic diagram of the formation of SEI. f) Active Li loss. g) Li residual and the structural defects of graphite.

Taking the currently popular NCM materials as an example and the reference of Li‐ion battery cathode materials, their structural degradation can be summarized into three aspects: active Li loss, irreversible structural transitions, and microcrack formation, as depicted in **Figure** **6**a,b,f. Additionally, despite the separator, the degradation of the anode and cathode are closely connected. In degraded graphite anodes, structural breakdown and layer separation occur over repeated charge‐discharge cycles. This happens because repeated Li^+^ deintercalation causes volume changes in the graphite, leading to mechanical stress and eventual structural damage.^[^ ^5^ ^]^ The associated internal stress occurs on the graphite lattice. The accumulated stress gradually causes the graphite particles to break, crack, and split.^[^ ^44^ ^]^ The residual Li intercalated within the graphite interlayers is not confined to the surface but extends into the bulk of the graphite particles, thereby inducing the formation of structural defects.^[^ ^45^ ^]^ Furthermore, upon cycling, the edges and surfaces of the graphite particles undergo destabilization.^[^ ^46^ ^]^ The formation of the SEI layer is an inevitable consequence that contributes to an increase in the electrical impedance (Figure 6c,d,e). Lithiated graphite is highly reactive, making it difficult to handle and reuse directly, as it can combust when exposed to air due to its Li content. Although graphite is cost‐effective and abundant, its production has a significant carbon footprint, requiring recycling to support a sustainable, low‐carbon economy. For degraded cathode materials, performance loss mainly stems from inactive Li and irreversible structural changes (Figure 6f,g). The Li loss mainly stems from the initial formation of SEI layer on anode surface, forming inactive Li species so‐called inactive Li.^[^ ^47^ ^]^ These inactive Li hardly return to the cathode, leading to increasing SEI resistance and ohmic resistance.^[^ ^47^ ^]^ Consequently, the continuous loss of active Li^+^ reduces battery capacity. Studies show that NCM523 particles lose ≈22% of their Li by the end of their lifespan compared to their original state. This Li loss triggers irreversible structural changes, causing permanent phase transformations in the cathode's crystal structure. Additionally, oxygen evolution reactions on the crystal surface further degrade the material.^[^ ^48^ ^]^ NCM will spontaneously reduce Ni^4+^ to Ni^2+^ and release oxygen at the same time, and this oxygen evolution will form a spinel phase or a rock salt phase on the surface (e.g., NiO), increasing the impedance. Additionally, the anisotropic volume change produces internal stress to form cracks.^[^ ^49^ ^]^ The microcracks within cathode secondary particles precipitate particle disintegration and may culminate in the loss of electrical contact, rendering certain regions of the active material inactive.^[^ ^50^ ^]^ Concurrently, phase transitions induce substantial and non‐uniform lattice contractions, which in turn generate internal stresses that nucleate microcracks emanating from the particle core.^[^ ^51^ ^]^ Furthermore, the ionic radius of Ni^2+^ (0.69 Å) and Li^+^ (0.76 Å) are sufficiently like facilitate cation intermixing during de‐lithiation, potentially obstructing Li^+^ diffusion pathways. This intermixing can impede Li^+^ transport, leading to diminished diffusivity and heightened mechanical stress, which in turn increases polarization. Consequently, the cell is more susceptible to reaching the threshold voltage during charge‐discharge cycles.^[^ ^52^ ^]^ The synergistic influence of these factors can worsen the degradation of cell performance.

The degradation mechanism of LFP is similar to that of NCM materials and is also affected by chemical stress factors,^[^ ^53^ ^]^ such as the growth of SEI, electrolyte decomposition, Li coating, or particle cracks. After the long‐term cycle of LFP, the Li in LFP batteries will gradually lose, resulting in capacity attenuation, but this loss is still different from NCM, this loss is usually caused by Li inventory loss (LLI), that is, Li^+^ cannot normally move between the positive and negative electrodes in the charge and discharge cycle. Li loss can also lead to the formation of Fe (III) phase, which can cause some Li‐Fe antistites defects.^[^ ^24^, ^54^ ^]^

Note: The anode and cathode materials in the two images were taken from the same battery and examined by the S4800‐4039 SEM on the same day.

#### 2.2.2 Degradation of Shell, Separator and Electrolyte

While this study may not delve into a detailed discussion of the other components, their degradation can also impact battery functionality. Battery shell degradation also critically impacts performance, whose key factors include mechanical stresses (vibration/impact/compression) causing deformation or fractures, repeated electrode volume changes during cycling inducing shell strain, corrosive electrolyte byproducts from decomposition and environmental exposure‐plastic warping at high temperatures or metal corroding in humidity. These combined stresses necessitate robust shell designs resilient to both internal and external degradation mechanisms.^[^ ^55^ ^]^

Separator breakdown is also one of the key factors contributing to cell and battery malfunctions. In the worst‐case scenario, a failed separator could initiate a thermal runaway.^[^ ^56^ ^]^ These components are engineered to withstand thermal, mechanical, and electrical stress without breaking down. Nevertheless, under conditions of severe thermal, mechanical, or electrical stress, the separator might fail.^[^ ^57^ ^]^ Thermal‐high temperatures cause shrinkage (e.g., polyethylene) or decomposition, compromising integrity and risking short circuits.^[^ ^58^ ^]^ Mechanical‐electrode volume changes stress the separator, damaging pore structure and weakening it over cycles, increasing rupture risk.^[^ ^59^ ^]^ Finally, chemical‐electrolyte reactions form pore‐blocking deposits, impairing ion conductivity.^[^ ^60^ ^]^

The degradation of electrolytes is primarily influenced by chemical factors. Above 60 °C, electrolyte thermal degradation occurs as organic solvents and Li salts break down, generating by‐products that accelerate further decomposition^[^ ^61^ ^]^. Simultaneously, electrochemical degradation occurs at electrode surfaces through SEI formation consuming electrolyte components, solvent decomposition via polymerization/ester exchange, and LiPF_6_ hydrolysis with trace water, producing corrosive HF that damages battery components.^[^ ^61^, ^62^ ^]^

## 3 Direct Regeneration and Operation Mechanism of Eutectic Molten Salt Method

**Figure 7:**
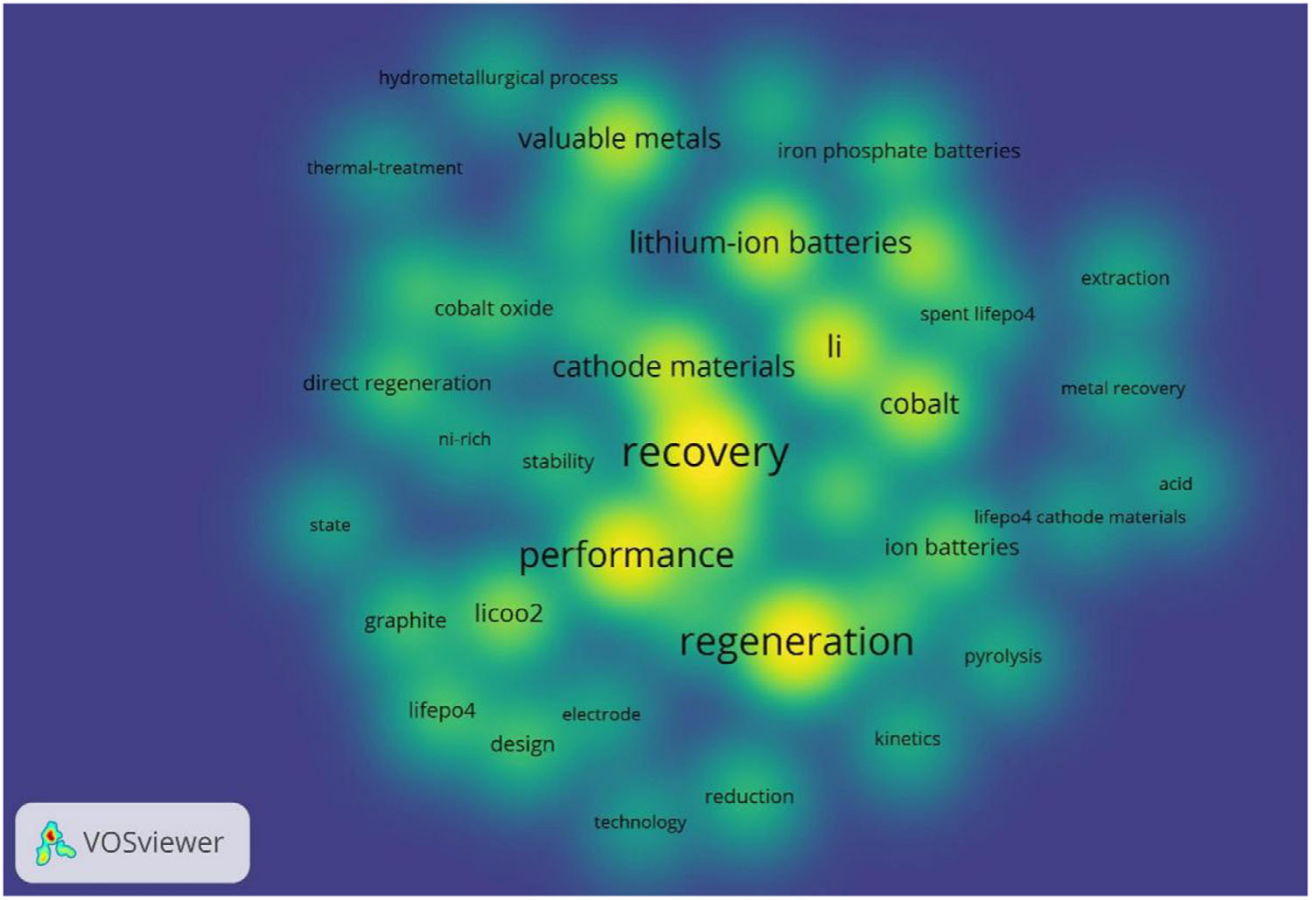
Direct regeneration heat map (Visualization tool: VOSviewer, Search criteria: Web of Science with “direct regeneration” and “cathode” as keywords and year of publication ranges from 2019 to 2024.).

Rejuvenating spent electrode materials through direct regeneration is a practical approach. This technique restores battery performance by adding back active elements and fixing structural issues, all without disassembling the inherent material structure.^[^ ^63^ ^]^ It's an eco‐friendly recycling method that conserves energy and minimizes environmental impact. In Li‐ion battery recycling, it's especially effective for reclaiming cathode materials.^[^ ^63^, ^64^ ^]^ As research into direct regeneration grows (which can refer to **Figure** **7**), a range of practical techniques has emerged, continually being optimized and innovated.

The high‐temperature solid‐phase technique combines used cathode materials with Li salts and subjects them to high‐temperature sintering, effectively restoring their structure and rectifying degraded characteristics.^[^ ^63^, ^65^ ^]^ The hydrothermal process, a favorite for treating LiFePO_4_, pushes Li back into the material under heat and pressure.^[^ ^66^ ^]^ The electrochemical method uses an electric field to re‐embed Li^+^, is simple, and eco‐friendly.^[^ ^67^ ^]^ The chemical re‐lithiation strategy boosts Li flow by oxidizing transition metals, healing the cathode in the process.^[^ ^68^ ^]^ Moreover, the solvothermal technique, which employs solvents under heat and pressure, can also replenish Li and heal the material's structure. By tweaking the reaction conditions, it allows for precise control over crystal development, shape, and properties.^[^ ^69^ ^]^ Speaking of efficiency, the eutectic molten salt method shines in restoring cathode materials, often using mixtures like LiNO_3_/LiOH.^[^ ^63^, ^70^ ^]^

Of all these, the eutectic molten salt method has captured significant attention as a novel and much‐debated direct regeneration technique. Understanding its mechanisms is key to improving the process and creating high‐quality, high‐value end products.^[^ ^23b^ ^]^

As the name suggests, the eutectic molten salt method involves treating spent cathode materials by utilizing a mixed salt system that melts above the eutectic temperature, thereby achieving regeneration. When the components of this mixed system adhere to specific molar ratios, the resulting eutectic molten salt exhibits a melting point lower than that of its individual constituents, making low melting temperature a defining characteristic of eutectic molten salt.^[^ ^71^ ^]^ Moreover, eutectic molten salt exhibits high energy storage density, low cost, and excellent chemical stability.^[^ ^72^ ^]^

Initially, eutectic molten salt was primarily employed in industrial applications. For instance, the NaNO_3_/KNO_3_ binary system, with a melting point of ≈220 °C, has been widely utilized as a thermal energy storage medium in concentrated solar power (CSP) systems.^[^ ^73^ ^]^ It was not until 2019 that Shi et al. achieved the first direct regeneration of Li‐ion battery NCM cathode materials under ambient pressure conditions. By employing a Li‐containing eutectic molten salt as the Li source to replenish Li in degraded cathodes, accompanied by brief sintering, they successfully restored the Li content, bulk/surface crystal structure, and electrochemical performance of the degraded materials to their pristine state. This milestone represented a significant breakthrough in applying eutectic molten salt to LIB recycling.^[^ ^74^ ^]^

From a comprehensive perspective, eutectic molten salt employed for LIB cathode material regeneration typically incorporates Li salts. During the repair process, these molten salts liquefy at temperatures exceeding their melting point, releasing Li ions to create a Li‐rich environment. The liquid molten phase simultaneously enhances Li‐ion diffusion kinetics, facilitating the migration of Li ions into the lattice structure of spent cathodes to replenish Li vacancies. Furthermore, the eutectic molten salt system undergoes thermochemical reactions during heating, which promote particle recrystallization of the cathode materials. This effectively eliminates structural defects such as lattice distortions and microcracks.^[^ ^71^, ^74^ ^]^ Collectively, these microscopic mechanisms establish optimal conditions for cathode regeneration.

So firstly, the mechanism of dissolution and recrystallization is a critical aspect. The eutectic molten salt method's effectiveness stems from its capacity to dissolve and recrystallize cathode materials, a key step in the process. This technique uses the liquid phase environment of molten salt to aid in the dissolution and recrystallization of materials like LiNi_0.5_Co_0.2_Mn_0.3_O_2_ or LiCoO_2_. When heated to temperatures between 400 to 850 °C, these cathode materials interact with salts such as LiOH, LiNO_3_, and Li_2_CO_3_,^[^ ^75^ ^]^ allowing Li^+^ to reintegrate into the material's lattice, compensating for the Li lost during battery use.^[^ ^75^, ^76^ ^]^ This phenomenon occurs via a solution‐recrystallization mechanism that enables both repair and regeneration of the cathode material's structure. Then comes phase transition and structural recovery, where the eutectic molten salt method not only supplements Li^+^ but also promotes the phase transition of the cathode material by adjusting reaction conditions (such as temperature and salt composition). For materials such as LiNi_0.5_Co_0.2_Mn_0.3_O_2_, the rock salt phase, and spinel phase can be transformed back to the original layered phase after molten salt treatment, thus restoring its electrochemical performance.^[^ ^75^, ^76^, ^77^ ^]^ Studies have shown that post‐salt treatment, a cathode material can have its phase impurities purged, and its layered structure restored through thermal treatments, like annealing at 850 °C.^[^ ^76^, ^77^ ^]^ Finally, another key mechanism of the eutectic molten salt method is to enhance the diffusion rate of Li^+^. The salts used have low melting points and are excellent conductors of ions, which speeds up the movement of Li^+^ within the material during the regeneration process. The introduction of Ca^2+^, for example, can boost the regeneration process and facilitate the transfer of Li^+^, creating an efficient diffusion pathway.^[^ ^75a^ ^]^

Additionally, the eutectic molten salt approach to cathode material regeneration presents distinct benefits over conventional recycling techniques. This method is characterized by reduced energy expenditure and lower emissions of noxious gases, offering a more sustainable alternative to pyrometallurgical and hydrometallurgical processes.^[^ ^75^, ^78^ ^]^ It facilitates the restoration of Li content and the reconstitution of cathode materials at lower thermal thresholds, thereby minimizing the potential for structural degradation associated with higher temperature treatments.^[^ ^76^, ^77^ ^]^ Regenerated cathode materials, when processed through the eutectic molten salt method, have demonstrated electrochemical properties comparable to those of newly manufactured materials. This method also conserves resources and presents economic advantages over traditional recycling methods.^[^ ^75^, ^77^ ^]^ Furthermore, the eutectic molten salt method exhibits broad applicability, being effective for an array of layered cathode materials.^[^ ^75^, ^78^, ^79^ ^]^

It is worth noting that in recent years, with the pursuit and exploration of eutectic molten salt system, the operation mechanism of molten salt method has been supplemented and updated. By changing the proportion of mixed molten salt, the melting point of the molten salt system is lower than the melting point of any single molten salt in the system, so the operating conditions of the eutectic molten salt method are more moderate. There will be two to three kinds of molten salt in the system most of the time.^[^ ^71^, ^75^, ^76^ ^]^

### 3.1 Comparison of Pyrometallurgical and Hydrometallurgical Methods and Direct Regeneration

To further highlight the advantages of direct regeneration, studies that employ direct regeneration methods for the recycling or recovery of battery cathode materials, with comparisons to traditional pyrometallurgical and hydrometallurgical processes have been collected. From these investigations, relevant information has been extracted regarding process costs, benefits, and greenhouse gas emissions, either from the main text or the supporting data. This information has been systematically organized and presented in **Table** **1**.

**Table 1:** Comparison of environmental friendliness and economic viability of pyrometallurgical, hydrometallurgical methods, and direct regeneration. C: cost ($ kg^−1^); P: product value ($ kg^−1^); G: greenhouse gas (GHG) emissions (g kg^−1^).

Cathode	Index	Pyrometallurgical method	Hydrometallurgical method	Direct regeneration
NCM111^[^ ^80^ ^]^	C	3.10	2.54	2.07
	P	2.16	3.07	4.85
	G	2457	2258	573
NCM523^[^ ^75b^ ^]^	C	5.15	7.85	6.25
	P	6.06	11.28	10.454
	G	N.A.	N.A.	N.A.
NCM523^[^ ^70a^ ^]^	C	3.102	2.692	2.668
	P	0.26	0.14	1.984
	G	14494	13828	9159
NCM523^[^ ^81^ ^]^	C^*)^	4.11	3.50	2.80
	P	N.A.	N.A.	N.A.
	G^*)^	3086	3328	2074
NCM523^[^ ^82^ ^]^	C	19.67	19.31	20.97
	P	6.51	5.33	6.75
	G	16165	14540	6716
LCO^[^ ^75a^ ^]^	C	N.A.	N.A.	N.A.
	P	12.09	12.23	17.28
	G	19300	17280	7820

*) Note: The original unit is $ kWh^−1^, which is also retained in the analysis in the paper. To maintain the rigor of the table, the cathode material required for a 1 kWh ternary battery was normalized according to the Ningde Era EIA Report (in Chinese), which is ≈19.756 kg (1975.6 tons /1000GWh). Source was accessed on October 18, 2024: https://max.book118.com/html/2020/0308/8073014045002101.shtm

**Figure 8:**
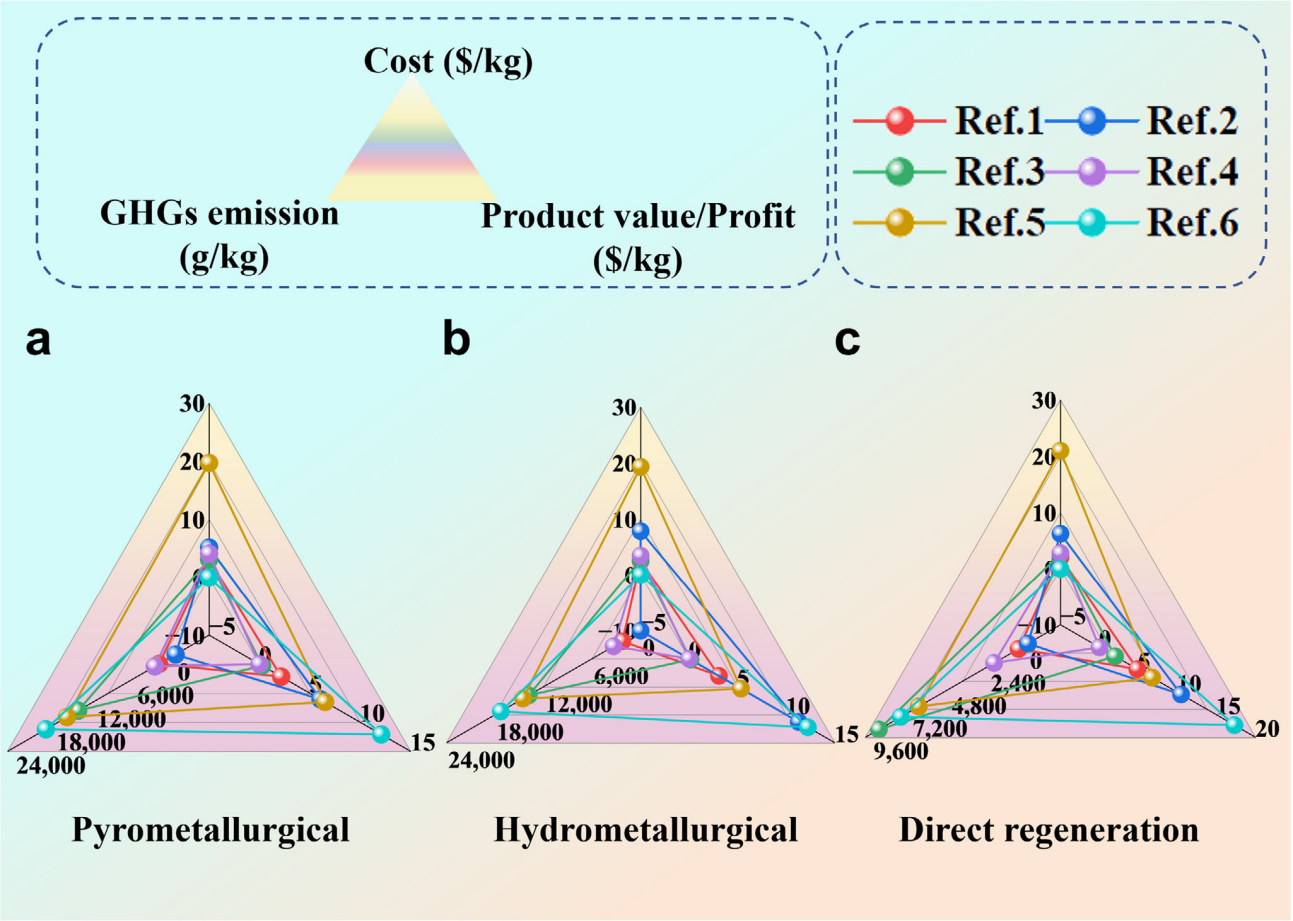
Comparison of environmental and economic friendliness, classified with three recycling methods: a) Pyrometallurgical method, b) Hydrometallurgical method, and c) Direct regeneration. The order of references corresponds sequentially to the top‐down listing in Table 1. Note: If an item of data in the image is not applicable, a value of 0 is assigned as the representative and maintains the normal composition of the image.

The comparative analysis of production costs and environmental impacts from the table underscores the economic and ecological advantages of direct regeneration methods over traditional pyrometallurgical and hydrometallurgical approaches. Despite the study by Wang et al., indicating a slightly higher production cost for direct regeneration at $20.97 per kilogram, compared to the conventional pyrometallurgical ($19.67 per kilogram) and hydrometallurgical ($19.31 per kilogram) methods, the economic benefit of the regenerated NCM material, at $6.75 per kilogram, surpasses the latter techniques.^[^ ^82^ ^]^ Consistently, direct regeneration often yields a higher product value or profit margin than its traditional counterparts. Exceptions occur where hydrometallurgical recycling may temporarily surpass direct regeneration in product value, potentially due to the inclusion of metal recovery values from conventional processes in the data. However, when noticing the high operational costs of hydrometallurgical methods and the comparatively lower costs of direct regeneration, the latter logically emerges as a more favorable option.^[^ ^75b^ ^]^ When assessing the environmental friendliness of direct regeneration processes based on greenhouse gas emissions, its superiority in this regard is unparalleled. In the studies that have been summarized, the direct regeneration process generates significantly fewer greenhouse gases compared to traditional pyrometallurgical and hydrometallurgical processes. Among them, from the absolute point of view, Yu et al. used the direct regeneration process to repair NCM111, only 573 g of greenhouse gases were emitted per kilogram of cathode material produced.^[^ ^80^ ^]^ Liu et al. found that the cost of manufacturing 1 kilowatt‐hour of NCM523 battery through pyrometallurgical recycling is ≈$81.25, while the hydrometallurgical process is $69.19, both substantially exceeding the direct regeneration cost of $55.35. Moreover, both traditional methods were responsible for over 60000 grams of greenhouse gas emissions, with the hydrometallurgical process standing out at 65747 grams.^[^ ^81^ ^]^ Encouragingly, recent advancements have refined the recycling process by incorporating calcium oxide to supply calcium ions, thereby enhancing the economic and environmental sustainability of the process. Gao et al. discovered that all three methods—pyrometallurgical, hydrometallurgical, and direct regeneration—yielded profits exceeding $10 per kilogram, with direct regeneration achieving a notably high profit of $17.28 per kilogram. Furthermore, the greenhouse gas emissions were as low as 7820 grams per kilogram.^[^ ^75a^ ^]^ More importantly, data review revealed no significant generation of solid or liquid waste during the direct regeneration process. In summary, direct regeneration emerges as a highly advantageous approach, combining strong economic benefits with a clear commitment to environmental sustainability, and **Figure** **8** can be used as a visual reference to illustrate this result concurrently.

## 4 Application and Improvement of Eutectic Molten Salt Method for Direct Regeneration

In recent years, the eutectic molten salt method has achieved remarkable advancements and transformative breakthroughs across several critical domains. Firstly, pretreatment technologies for battery regeneration have undergone substantial optimization, with significant progress realized in the efficient discharge, systematic disassembly, and precise stripping of cathode materials. Secondly, the development of novel molten salt systems has gained momentum, with extensive exploration into diverse combinations of eutectic molten salt to tailor their properties for specific applications. Thirdly, the regeneration process itself has been refined, particularly through the adjustment of key parameters such as temperature profiles. Additionally, substantial efforts have been directed toward enhancing process performance, particularly in maintaining and even augmenting the electrochemical properties of regenerated materials. Lastly, there is a concerted drive toward establishing greener and more economically viable process systems, aligning with global sustainability goals. These collective advancements underscore the method's potential to revolutionize battery recycling, offering a pathway to both environmental management and economic efficiency.

### 4.1 Pretreatment

Before the direct regeneration technology is implemented, the used batteries need to be discharged and disassembled to lower the operating risk. The Chinese national standard document “Cycle life requirements and test methods for traction battery of electric vehicle (GB/T 31484–2015)” is taken as a reference, commonly recommended discharge methods include constant discharge (CC), constant voltage discharge (CV), constant current–constant voltage discharge (CC–CV), constant power discharge (CP), constant power‐constant current‐constant voltage discharge (CP–CC–CV), constant resistance discharge (CR) and constant resistance‐constant voltage discharge method (CR–CV). The most common discharge mode of LIBs is CC, the current remains constant throughout the discharge process, the voltage gradually decreases to the termination voltage, and the discharge ends.^[^ ^83^ ^]^ Under normal circumstances, LIBs will relax after resting for a period after discharge, that is, voltage rebound. In recent years, Wang et al. have developed an effective and clean method for discharging old batteries, using salt solutions such as sodium chloride (NaCl), sodium carbonate (Na_2_CO_3_), and sodium sulfate (Na_2_SO_4_) to treat discharged batteries, effectively preventing voltage rebound, in which NaCl solution is the best treatment, while Na_2_CO_3_ solution treatment produces the least pollutants.^[^ ^84^ ^]^ Another study also proved that solution immersion discharge is feasible, and sodium hydroxide (NaOH) has also been developed and used and can keep the degree of corrosion of the battery at a low level.^[^ ^85^ ^]^ At the same time, sodium sulfide (Na_2_S), magnesium sulfate (MgSO_4_), and other solutions can also be used in the discharge process of LIBs, and the introduction of ultrasonic technology in the discharge process can improve the discharge efficiency, and the discharge time can be reduced by more than ten times.^[^ ^86^ ^]^ Copper sulfate solution (CuSO_4_) is also considered to be an efficient and green discharge medium, the shortest discharge time of spent LIBs in 5wt.% CuSO_4_ solution is 5 min, and the mobility of Li is as high as 60.83%.^[^ ^87^ ^]^ It is worth noting that spent batteries can be treated with liquid nitrogen to achieve freezing effects to avoid thermal runaway during discharge and transfer.^[^ ^88^ ^]^ In addition, liquid nitrogen can also be used as a physical discharge medium to prevent the combustion of electrolytes. The violent reaction of oxygen and nitrogen can also be avoided during the disassembly process.^[^ ^89^ ^]^ After the discharge is completed, the manual disassembly process can be safely done. A clear trend toward methodological diversification is evident in battery discharge processes, though salt solution‐based approaches remain predominant. Continuous optimization of salt solution formulations has not only reduced the environmental impact of discharge operations but also enhanced process efficiency while addressing the critical issue of voltage rebound. Emerging technologies such as liquid nitrogen treatment and ultrasound‐assisted discharge demonstrate unique advantages, particularly in thermal runaway suppression (enhancing safety) and significant discharge time reduction. These developments provide a diverse technological toolkit for LIB discharge pretreatment. However, achieving optimal balance between efficiency, safety, and environmental sustainability remains crucial. Future advancements should focus on improving the green chemistry and economic viability of discharge methods and developing integrated approaches (e.g., combining liquid nitrogen cryogenic treatment, salt solution discharge, and ultrasonic assistance) to overcome scalability challenges in industrial applications.

Then, what needs to be done is to separate the cathode material from the Al foil. Common methods are organic solvents, such as N, N‐dimethylformamide (DMF), N‐methyl pyrrolidone (NMP), and dimethyl sulfoxide (DMSO), which can be used to dissolve the adhesive polyvinylidene fluoride (PVDF), by soaking the positive electrode sheet in the above solution, heating and stirring. It can realize the separation of Al foil and cathode material.^[^ ^90^ ^]^ Although these solvents can bring good stripping effect, but in the separation process will produce a lot of organic wastewater, and DMF, NMP, and so forth are also toxic, easy to cause environmental impacts and human health effects.^[^ ^91^ ^]^ Moreover, NMP has a high boiling point, and even though most of the NMP material will evaporate during the cathode material baking process, trace residues occur in the cathode containing nano‐scale and high‐porosity active materials.^[^ ^92^ ^]^ In fact, the organic solvent method is also being improved and improved, for example, triethyl phosphate (TEP) is considered to be a good alternative to DMF, in addition to being non‐toxic and more environmentally friendly, TEP has a superior performance in stabilizing and swelling PVDF adhesives and it also has excellent thermal safety itself.^[^ ^93^ ^]^ Glycerin is also a kind of green organic solvent. When the heating temperature is 200 °C, the stirring speed is 350 r min^−1^, and the stirring time is 15 min, the stripping rate of Al foil and cathode material can reach 95.47%.^[^ ^94^ ^]^ More notably, the environmentally friendly and efficient green low eutectic solvent (DES) has also been developed for the stripping of Al foil and cathode materials, at 120 °C, with choline chloride and ethylene glycol in a 1:2 molar ratio of eutectic solvent treatment electrode sheet for 8 min, and the use of microwave assisted treatment, the stripping rate of cathode materials can reach 100%.^[^ ^95^ ^]^ Another eutectic solvent system is composed of low‐toxic, low‐cost choline chloride and glycerin at a molar ratio of 2.3:1, and heated at 190 °C for 15 min, helping obtain 99.86wt.% of the material stripping rate.^[^ ^96^ ^]^ In addition to the mentioned solvents, some molten salt systems have also been well applied in the stripping process of cathode materials and Al foil, for example, the molten salt system composed of AlCl_3_ and NaCl is not only non‐toxic, but also heated for 20 min at 160 °C with a mass ratio of molten salt to cathode material of 10:1, so that PVDF can be effectively melted, and the stripping rate can reach 99.8%.^[^ ^97^ ^]^ In recent years, the introduction of advanced oxidation processes has also brought a new dawn for the stripping of cathode materials and Al foil. An improved advanced oxidation system (UASF) uses peroxydisulfate (S_2_O_8_^2−^) and ferrous ion (Fe^2+^) as the catalyst for free radical production under ultrasonic assistance. The resulting sulfate free radical and hydroxyl free radical can oxidize PVDF. Thus, the stripping efficiency of the cathode material can reach 100%, and the reaction can be carried out at 35 °C.^[^ ^98^ ^]^ Oxalic acid, sulfuric acid, and other acid solutions can also strip off Al foil and cathode materials with the help of ultrasonic waves. When the temperature is 99 °C and the ultrasonic power is 98 W, sulfuric acid solution has the best stripping effect on cathode materials. When the temperature is 100 °C and the ultrasonic power is 99 W, oxalic acid solution has the best stripping effect on cathode materials, and both can strip off the cathode material quickly in a short time. And it can almost achieve the effect of complete stripping, which may be due to the violent oxidation of PVDF by hydroxyl free radicals generated in the system.^[^ ^99^ ^]^ Finally, emerging Joule thermal technology also brings a new idea for cathode material stripping. When the Al foil is injected with Joule heat directly in the air, PVDF can melt and slightly thermal decomposition, and the bonding performance is reduced and destroyed. The width and energy of the electrical pulse have a certain impact on the stripping effect, the experiment shows that the temperature of the Al foil is raised to ≈40K in 750 microseconds, the best separation effect can be achieved, and the material stripping rate is as high as 99%.^[^ ^100^ ^]^

The stripping system exhibits increasing diversification, evolving from conventional solvent‐based methods to modified DES, eutectic molten salts, advanced oxidation processes, and emerging joule heating techniques—each offering distinct advantages for cathode material stripping. However, method selection requires careful consideration of multiple factors, including efficiency, economic viability, environmental impact, and operational safety. Each approach presents inherent limitations: organic solvents may pose toxicity concerns, eutectic molten salt can demonstrate corrosive behavior, advanced oxidation processes often involve costly oxidants, and joule heating demands precise technical control. Given these trade‐offs, future efforts toward scalable stripping should explore hybridized systems that integrate complementary technologies, mitigating individual shortcomings while maximizing overall performance. Such synergistic strategies could enhance both sustainability and practicality in industrial‐scale battery recycling.

### 4.2 Application of Eutectic Molten Salt Method for Direct Regeneration of Libs

In recent years, the eutectic molten salt method has developed rapidly and has been successfully used to directly regenerate various cathode materials. As a result, case studies of the eutectic molten salt method have been gathered and organized in **Table** **2**. It is clear from Table 2 that the eutectic molten salt method is highly adaptable. Researchers have used different combinations of molten salt, their respective ratios, and various calcination and annealing temperatures to directly regenerate different degraded cathode materials, achieving very promising repair outcomes. The results show that batteries made from cathode materials regenerated using the molten salt method exhibit good cycling stability. After 100 or more cycles, those regenerated batteries’ capacity retention all remains above 80% and mostly remains >90%.

**Table 2:** Direct regeneration of various cathode materials with eutectic molten salt method.

Eutectic mixture (Molar ratio)	Temperature and duration	Capacity retention [Cycle number]	Rate	Refs.
LFP				
LiNO_3_:FeC_2_O_4_ 5:1	300 °C for 2 h 650 °C for 6 h	95.0% (100)	0.5C	[101]
LiNO_3_:LiOH 3:2	550 °C for 4 h	97.73% (150)	1C	[102]
NCA				
LiOH:Na_2_SO_4_ 5:1	750 °C for 15 h	85.1% (250)	1C	[103]
NCM811				
LiOH:NaCl 5:1	850 °C for 15 h	86.5% (200)	1C	[104]
NCM622				
LiOH:Li_2_SO_4_ 6:1	900 °C for 20 h	99.4% (250)	1C	[76b]
LiNO_3_:LiOH 3:2	500 °C for 5 h 850 °C for 11 h	94.3% (240)	1C	[105]
NCM523				
LiNO_3_:LiOH 3:2	300 °C for 4 h 850 °C for 4 h	90.2% (100)	1C	[74]
LiOH:LiI 3:1	200 °C for 4 h 850 °C for 5 h	80.0% (200)	0.5C	[71]
LiOH:LiNO_3_:LSA 2:3:5	350 °C for 4 h 850 °C for 6 h	95.6% (100)	0.5C	[75b]
LiOH:Li_2_CO_3_ 43:7	440 °C for 5 h 850 °C for 12 h	89.06% (200)	1C	[77]
LiOH:Li_2_CO_3_ 42:8	450 °C for 9 h 700 °C for 2h	92.0% (100)	1C	[106]
KCl:KNO_3_:LiNO_3_ 17:14:2	750 °C for 12h	95.5% (100)	0.2C	[107]
LiOH:LiNO_3_:CH_3_COOLi 6:9:10	400 °C for 4 h 850 °C for 5 h	93.7% (100)	0.5C	[76a]
LiOH:Li_2_CO_3_:LiNO_3_ 2:1:1	500 °C for 5 h 950 °C for 10 h	91.7% (100)	1C	[108]
LCO				
LiOH:KOH 3:7	300 °C for 8 h 500 °C for 16 h	93.0% (100)	0.2C	[109]
LiNO_3_:KCl 2:3	750 °C for 12 h	95.6% (150)	0.5C	[75a]
LiOH:KOH:Li_2_CO_3_ 6:14:1	500 °C for 8 h	92.5% (200)	0.2C	[110]

Note: LSA is lithium salicylate

#### 4.2.1 Direct Regeneration of LFP with Eutectic Molten Salt Method

**Figure 9:**
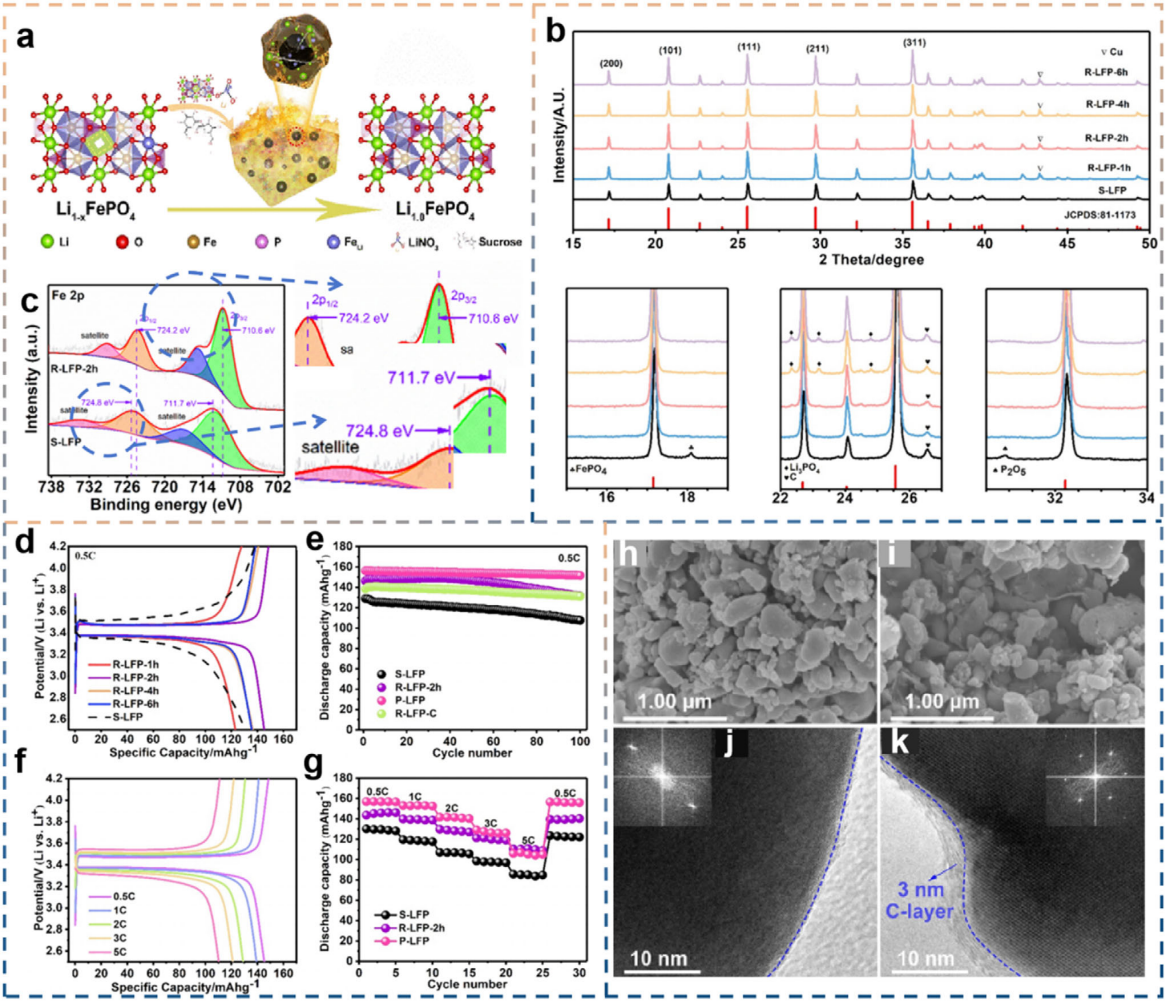
Direct regeneration of LFP with LiNO_3_ and FeC_2_O_4_. a) Schematic diagram of structural restoration of degraded LFP. Reproduced with permission.^[^ ^101^ ^]^ Copyright 2022, American Chemical Society. b) XRD patterns of spent LFP (S‐LFP) and regenerated LFP treated in 1, 2, 4, and 6 h. Reproduced with permission.^[^ ^101^ ^]^ Copyright 2022, American Chemical Society. c) XPS pattern of S‐LFP and R‐LFP‐2 h. Reproduced with permission.^[^ ^101^ ^]^ Copyright 2022, American Chemical Society. d) Charge−discharge curves at 0.5C. Reproduced with permission.^[^ ^101^ ^]^ Copyright 2022, American Chemical Society. e) Cycling performance at 0.5C. f) Charge−discharge curves of R‐LFP‐2 h at different rates. Reproduced with permission.^[^ ^101^ ^]^ Copyright 2022, American Chemical Society. g) Rate performance of S‐LFP, R‐LFP‐2 h, and P‐LFP. Reproduced with permission.^[^ ^101^ ^]^ Copyright 2022, American Chemical Society. h) SEM image of S‐LFP. Reproduced with permission.^[^ ^101^ ^]^ Copyright 2022, American Chemical Society. i) SEM image of R‐LFP‐2 h. Reproduced with permission.^[^ ^101^ ^]^ Copyright 2022, American Chemical Society. j) TEM image of S‐LFP. Reproduced with permission.^[^ ^101^ ^]^ Copyright 2022, American Chemical Society. k) TEM image of R‐LFP‐2 h. Reproduced with permission.^[^ ^101^ ^]^ Copyright 2022, American Chemical Society.

Although there aren't many reports on the direct regeneration of LFP using eutectic molten salt, existing studies show that the results are quite promising. For example, batteries regenerated with a combination of LiNO_3_ and FeC_2_O_4_ maintain a capacity retention of 95% after 100 cycles at 0.5C.^[^ ^101^ ^]^ Similarly, those regenerated using LiNO_3_ and LiOH achieve a high capacity retention of 97.73% after 100 cycles at 1C.^[^ ^102^ ^]^ Take direct regeneration in a reductive environment as an example, the reductive environment prevents the conversion of Fe^2+^ to Fe^3+^, the molten salt completes the replenishment of Li^+^ for the degraded LFP, and the subsequent annealing restores the structure of the LFP (**Figure** **9**a). X‐ray diffraction (XRD) analysis revealed that the regenerated LFP (R‐LFP) exhibited a more favorable crystal orientation with enhanced exposure of (101) planes (Figure 9b). In particular, the (101) crystal plane diffraction peak intensity of R‐LFP‐2 h sample is the strongest, indicating that the sample exposes more (101) crystal faces, which is conducive to the diffusion of Li^+^. X‐ray Photoelectron Spectroscopy (XPS) results confirmed the reduction of Fe^3+^ to Fe^2+^, indicating effective structural repair (Figure 9c). The regenerated LFP achieved a specific capacity of 145 mAh g^−1^ at 0.5C, a 13% improvement over spent LFP, and demonstrated superior rate performance compared to pristine LFP (Figure 9e). Although R‐LFP‐2 h has a slightly lower capacity than the original LFP at low magnification, it performs better at high magnification, which is consistent with the (101) crystal face exposed in the XRD analysis and the optimized Li‐ion diffusion channel (Figure 9g). SEM (Figure 9h,i) and transmission electron microscope (TEM) (Figure 9j,k) analyses showed that the regeneration process eliminated surface defects and improved crystallinity, with a uniform carbon coating enhancing conductivity.

The study indicates that the regenerated LFP exhibits increased exposure of (101) crystal planes, which facilitates Li^+^ diffusion. However, the influence of different crystal facets (e.g., (010), (001) on electrochemical performance may vary. This finding provides insights into future optimization of Li^+^ diffusion pathways. Potential strategies may involve selective exposure of specific crystallographic planes through molten salt composition design (e.g., incorporation of tailored surfactants) or dynamic annealing processes (temperature gradient modulation),^[^ ^111^ ^]^ thereby enabling precise regulation of Li replenishment and minimizing Li resource wastage. Furthermore, SEM/TEM analysis reveals improved homogeneity of the carbon coating in regenerated samples, which may contribute to enhancing the conductivity of LFP.^[^ ^112^ ^]^ Future investigations could explore the carbon source—if substantial carbon residues persist on spent LFP surfaces,^[^ ^113^ ^]^ such inherent carbon might serve as a reducing medium to stabilize iron oxidation states, thereby reducing the consumption of reductive Li salts.

#### 4.2.2 Direct Regeneration of NCA with Eutectic Molten Salt Method

**Figure 10:**
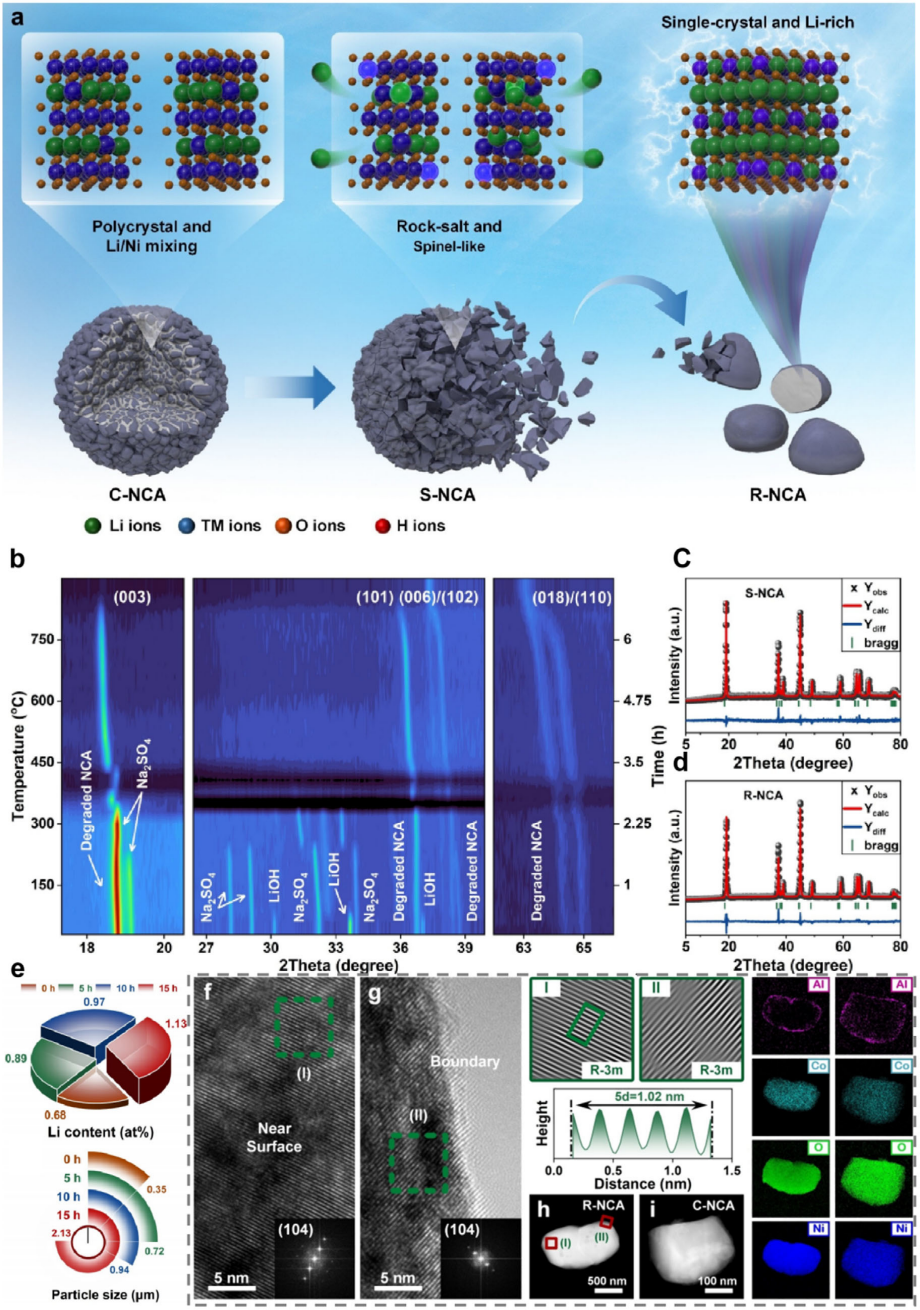
Direct regeneration of NCA with LiOH and Na_2_SO_4_. a) Schematic diagram of structural restoration of degraded NCA. Reproduced with permission.^[^ ^103^ ^]^ Copyright 2023, Wiley‐VCH. b) Contour plot of the in situ XRD patterns recorded during the eutectic molten salt treatment. Rietveld refinement results of c) S‐NCA and d) R‐NCA. Reproduced with permission.^[^ ^103^ ^]^ Copyright 2023, Wiley‐VCH. e) ICP‐OES and particle size analysis with different treatment durations. Reproduced with permission.^[^ ^103^ ^]^ Copyright 2023, Wiley‐VCH. HR‐TEM image of bulk f) and boundary g) in R‐NCA. The inserts show the fast Fourier transform (FFT) images of the areas marked by dashed boxes. Reproduced with permission.^[^ ^103^ ^]^ Copyright 2023, Wiley‐VCH. The images labeled (I) and (II) on the right are the corresponding inverse FFT images, with schematic diagrams of interplanar spacing provided below. STEM of h) R‐NCA and (i) C‐NCA with the EDS mapping distribution of O, Ni, Co, and Al elements. Reproduced with permission.^[^ ^103^ ^]^ Copyright 2023, Wiley‐VCH.

Similarly, reports on the use of eutectic molten salt methods for NCA regeneration are relatively limited, but their effectiveness is undeniable. A combination of LiOH and Na_2_SO_4_ in a 5:1 ratio, when heated at 750 °C, can achieve the most optimal regeneration results for spent NCA (S‐NCA). The regenerated NCA (R‐NCA) batteries retain 85.1% of their capacity after 250 cycles at 1C.^[^ ^103^ ^]^ This method not only reduces the degree of Li/Ni disorder of NCA but also eliminates the rock salt phase on its surface and finally obtains the single‐crystalline layered NCA rich in Li (**Figure** **10**a). The in situ XRD patterns (Figure 10b) reveal the structural evolution from amorphous to crystalline states as temperature increases. The disappearance of peaks at 350 °C and their re‐emergence at 450 °C indicate a phase transition associated with the melting and recrystallization process. At higher temperatures, the characteristic peak of (003) is gradually enhanced, indicating that the crystal structure of the material is gradually restored and rebuilt. This process confirmed that molten salt treatment can effectively repair the crystal structure of S‐NCA and promote the replenishment and redistribution of Li. Rietveld refinement (Figure 10c,d) further elucidates the Li/Ni cation mixing in S‐NCA and R‐NCA, showing that the molten salt treatment not only replenishes Li but also forms a Li‐rich structure by inserting excess Li into the Ni layers, which is crucial for enhancing electrochemical performance. Inductively coupled plasma‐optical emission spectrometer (ICP‐OES) and particle size analysis explain that the Li content and particle size increase with prolonged treatment time, indicating effective Li supplementation and secondary grain growth (Figure 10e). High resolution transmission electron microscope (HR‐TEM) images confirm the single‐crystalline nature and consistent lattice parameters of R‐NCA, with uniform interplanar spacings indicative of a well‐reconstructed layered structure (Figure f, g). Scanning transmission electron microscopy (STEM) and energy dispersive X‐ray spectrometer (EDS) mapping demonstrate the homogeneous distribution of elements in R‐NCA, highlighting the successful structural repair and element redistribution which is similar to commercial NCA (C‐NCA) (Figure 10h,i). STEM images show that R‐NCA particles have no distinct grain boundaries within them, indicating their single‐crystal properties. The EDS element distribution diagram shows that O, Ni, Co, and other elements are evenly distributed, while Al elements tend to concentrate on the particle surface.

The study demonstrates that eutectic molten salt enables precise elemental regulation during the direct regeneration of spent NCA cathodes, achieving uniform Ni/Co distribution while inducing Al surface segregation. This suggests that molten salt not only replenishes Li but also modulates elemental positioning. Notably, the observed Al surface segregation may lead to the in situ formation of an Al_2_O_3_ layer, which could potentially mitigate electrolyte corrosion.^[^ ^114^ ^]^ This finding offers an intriguing perspective: future studies might explore the intentional addition of tailored Al sources into molten salt to directionally construct core‐shell structures, thereby enhancing electrode protection.^[^ ^115^ ^]^

#### 4.2.3 Direct Regeneration of NCM811 with Eutectic Molten Salt Method

**Figure 11:**
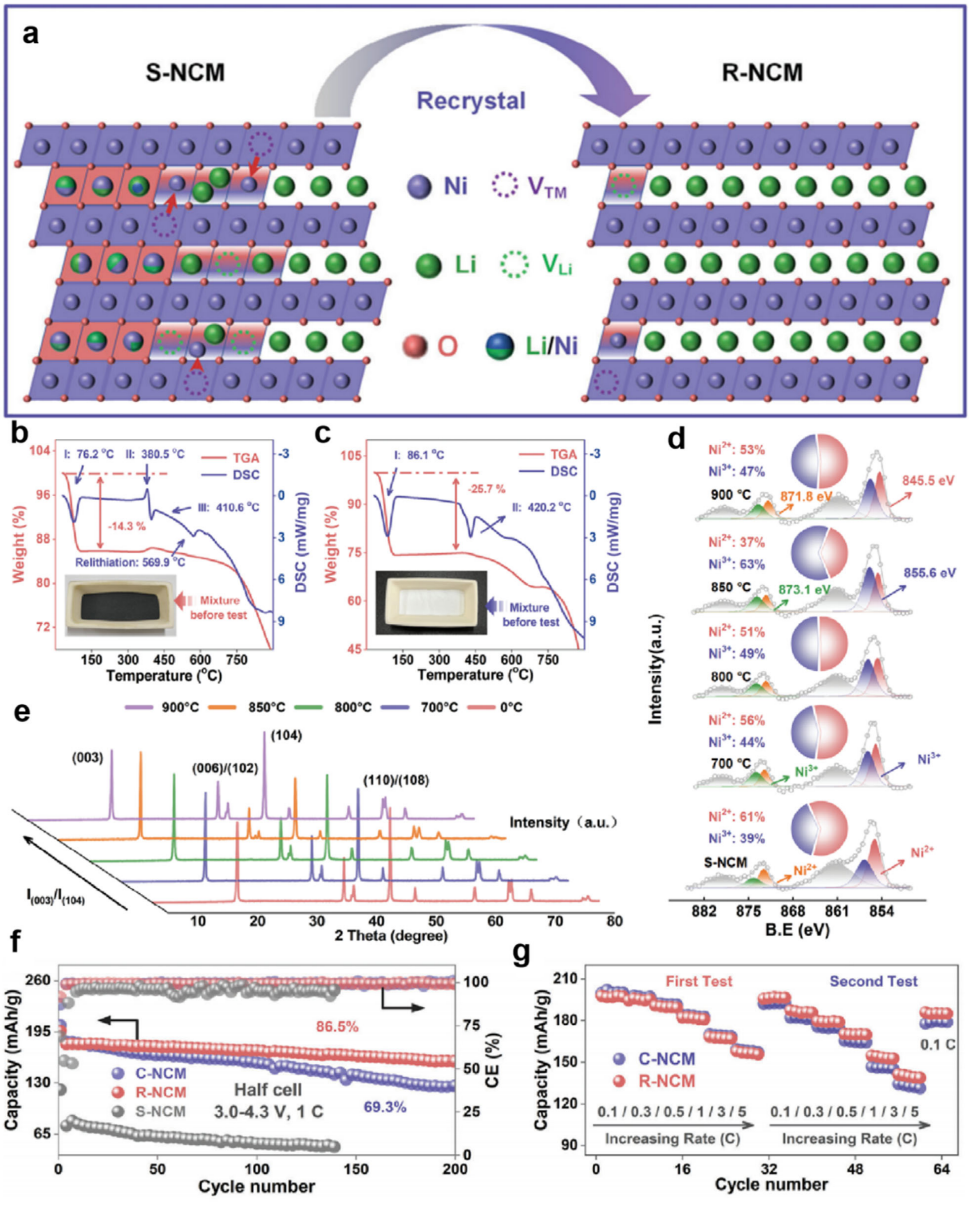
Direct regeneration of NCM811 with LiOH and NaCl. a) Schematic illustration of the microstructural transformation from S‐NCM to R‐NCM. Reproduced with permission.^[^ ^104^ ^]^ Copyright 2023, Wiley‐VCH. b) DSC and TGA curves of S‐NCM, LiOH, and NaCl from 30 to 900 °C. Reproduced with permission.^[^ ^104^ ^]^ Copyright 2023, Wiley‐VCH. c) DSC and TGA curves of LiOH and NaCl from 30 to 900 °C. Reproduced with permission.^[^ ^104^ ^]^ Copyright 2023, Wiley‐VCH. d) XPS spectra for Ni 2p of S‐NCM and R‐NCM in treating temperatures of 700, 800, 850, and 900 °C, respectively. Reproduced with permission.^[^ ^104^ ^]^ Copyright 2023, Wiley‐VCH. Electrochemical performance assessed within a voltage window of 3.0–4.3 V via half‐cell tests: f) Long‐duration cycling at a rate of 1C (1C = 150 mA g^−1^), and g) rate performance test. Reproduced with permission.^[^ ^104^ ^]^ Copyright 2023, Wiley‐VCH.

The performance of the eutectic molten salt method in the direct regeneration of NCM811 remains remarkable. After 200 cycles at a 1C rate, the NCM811 battery regenerated using the LiOH/NaCl molten salt system still maintains a capacity retention rate of 86.5%.^[^ ^104^ ^]^ And the successful recrystallization confirms the Li replenishment and the alleviation of Li/Ni disorder (**Figure** **11**a). During thermogravimetric analysis (TGA) and Differential scanning calorimetry (DSC) testing, the TGA curve shows significant weightlessness below 100 °C, which may be due to the dehydrating effect of Li salts (Figure 11b). In the DSC curve, the endothermic peak near 415 °C is consistent with the eutectic point of the LiOH/NaCl system, indicating that the molten salt begins to melt at this temperature, providing conditions for the subsequent Li^+^ replenishing and phase reconstruction. Figure 11c further confirms the eutectic point of the LiOH/NaCl system at around 415 °C, providing a critical molten salt environment for the subsequent regeneration process. Figure 11d shows the Ni 2p XPS spectra, indicating a gradual reduction of Ni^2+^ proportion from 700 to 850 °C, suggesting the recovery of the layered structure as Li ions are re‐embedded. However, at 900 °C, the increase in Ni^2+^ suggests Li loss due to excessive volatilization. At the same time, the intensity ratio of (003) and (104) peaks (I(003)/I(104)) is an important index to evaluate the quality of the crystal structure of the material. The I(003)/I(104) ratio increased with the increase of the treatment temperature (Figure 11e), indicating that the crystal structure of spent NCM (S‐NCM) gradually recovered well. When the temperature reaches 900 °C, a sudden decrease in the I(003)/I(104) ratio may imply another serious degradation of the crystal structure, which echoes the increase in the Ni^2+^ ratio in the XPS graph. The cycling performance test presented in Figure 11f demonstrates that the regenerated NCM (R‐NCM) exhibits remarkable cycling stability, maintaining a high capacity retention rate over multiple charge‐discharge cycles. This is crucial for the service life and performance of Li‐ion batteries. The rate performance indicates that, although the two materials show similar rate capabilities in the first test, R‐NCM delivers a higher capacity than commercial NCM (C‐NCM) in the subsequent second test as the current density increases (Figure 11g). This suggests that R‐NCM can still maintain good electrochemical performance at high current densities, thereby exhibiting superior rate performance. The study identifies the optimal temperature range for cathode regeneration within the LiOH/NaCl system, while also suggesting the need for future research to develop more diversified, energy‐efficient, and cost‐effective strategies to counteract Li volatilization. Notably, the regenerated NCM cathode exhibits higher capacity than commercial NCM under high‐rate cycling, raising intriguing questions regarding whether the eutectic molten salt with long‐range disordered structure enhances Li^+^ transport kinetics or inadvertently constructs more efficient ion‐conduction pathways during the regeneration process.^[^ ^116^ ^]^

#### 4.2.4 Direct Regeneration of NCM622 with Eutectic Molten Salt Method

**Figure 12:**
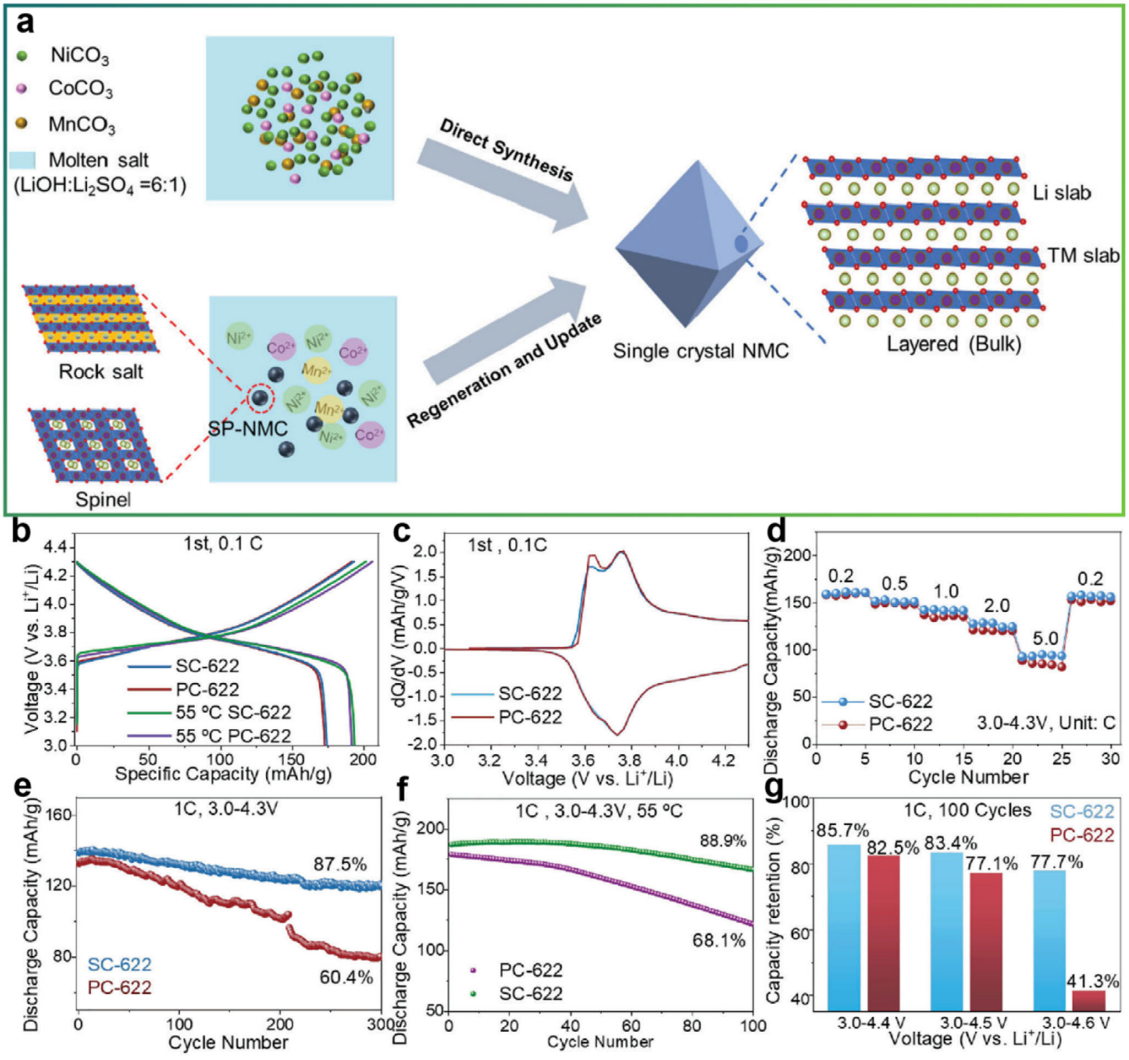
Direct regeneration of NCM622 with LiOH and Na_2_SO_4_. a) Schematic diagram of the procedures for fabricating single‐crystal NCMs via direct synthesis and the regeneration of spent NCM. Reproduced with permission.^[^ ^76b^ ^]^ Copyright 2024, Wiley‐VCH. b) Initial charge‐discharge profiles of SC‐622 and PC‐622 at ambient temperature and 55 °C. Reproduced with permission.^[^ ^76b^ ^]^ Copyright 2024, Wiley‐VCH. c) dQ/dV plots corresponding to the first cycle for SC‐622 and PC‐622. Reproduced with permission.^[^ ^76b^ ^]^ Copyright 2024, Wiley‐VCH. d) Rate capabilities of SC‐622 and PC‐622 between 3.0 and 4.3 V versus Li^+^/Li. Reproduced with permission.^[^ ^76b^ ^]^ Copyright 2024, Wiley‐VCH. e) Cycling behavior of SC‐622 and PC‐622 at 1C under ambient conditions. Reproduced with permission.^[^ ^76b^ ^]^ Copyright 2024, Wiley‐VCH. f) Cycling behavior of SC‐622 and PC‐622 at 1C at 55 °C. Reproduced with permission.^[^ ^76b^ ^]^ Copyright 2024, Wiley‐VCH. g) Cycling stability of SC‐622 and PC‐622 at various cutoff voltages. Reproduced with permission.^[^ ^76b^ ^]^ Copyright 2024, Wiley‐VCH.

The eutectic molten salt method has achieved remarkable success in the direct regeneration process of NCM622. Wang et al. employed a combination of LiOH and Li_2_SO_4_ to directly synthesize and regenerate single‐crystal NCM622 (SC‐622) in a one‐step process. The regenerated material, when assembled into batteries, exhibited an impressive capacity retention rate of 99.4% after 250 cycles at 1C, demonstrating excellent cycling stability.^[^ ^76b^ ^]^ This one‐step method not only simplifies the processing steps but also replenishes the Li vacancies in spent NCM622. The undesired rock‐salt phase is successfully eliminated, and the structure is effectively restored (**Figure** **12**a). The single‐crystal NCM622 treated with eutectic molten salt demonstrates superior electrochemical performance compared to its polycrystalline counterpart (PC‐622). SC‐622 exhibits a discharge capacity of 174 mAh g^−1^ at room temperature and 193 mAh g^−1^ at 55 °C, while PC‐622 shows similar voltage profiles. This similarity suggests that both materials have comparable initial electrochemical performance. However, the higher capacity at elevated temperatures for SC‐622 implies better kinetic properties at high temperatures (Figure 12b). Figure 12c shows the presence of distinct redox peaks in both materials, which indicates favorable electrochemical reversibility. But the redox peaks of PC‐622 degrade more severely with cycling compared to SC‐622, suggesting that SC‐622 has superior structural stability and resistance to degradation. Concurrently, the rate performance indicates that SC‐622 outperforms PC‐622 at all tested rates. A significant divergence is observed at 5C, where SC‐622 delivers a discharge capacity of 94.2 mAh g^−1^, whereas PC‐622 only achieves 85.6 mAh g^−1^ (Figure 12d). This enhancement is likely attributable to the single‐crystal structure of SC‐622, which facilitates the diffusion of Li^+^. More notably, SC‐622 exhibits significantly superior cycling stability under both room temperature and 55 °C conditions (Figure 12e,f). This characteristic also enables SC‐622 to be suitable for high‐temperature applications. In the cutoff voltage analysis, SC‐622 did not show a significant advantage over PC‐622 within the voltage ranges of 3.0–4.4 and 3.0–4.5 V. Under the voltage range of 3.0–4.6 V, SC‐622 achieved a capacity retention rate of 77.7%, while PC‐622 only maintained 41.3%, after 100 cycles at 1C (Figure 12g). This result indicates that SC‐622 can operate stably at higher voltages without significant capacity loss.

This study demonstrates that eutectic molten salt can simultaneously achieve Li replenishment, rock‐salt phase elimination, and single‐crystal transformation of spent NCM622 cathodes in a single‐step process, further confirming the efficiency and technical advantages of molten salt regeneration. Of particular interest is the incorporation of sulfate additives. Under high‐temperature conditions, the activated oxidative properties of sulfates may facilitate the oxidation and removal of surface rock‐salt phases,^[^ ^117^ ^]^ thereby promoting structural reconstruction of the degraded cathode material.^[^ ^118^ ^]^ Furthermore, it is scientifically valuable to investigate whether sulfur residues persist in the final product, and if the potential formation of Li–S–O passivation layers at the interface contributes to the observed stability enhancement.^[^ ^119^ ^]^ These considerations may be worth more investigation and provide fundamental mechanistic insights for future optimization of molten salt systems, potentially enabling more spent cathodes to achieve post‐regeneration performance comparable to commercial battery materials.

#### 4.2.5 Direct Regeneration of NCM523 with Eutectic Molten Salt Method

**Figure 13:**
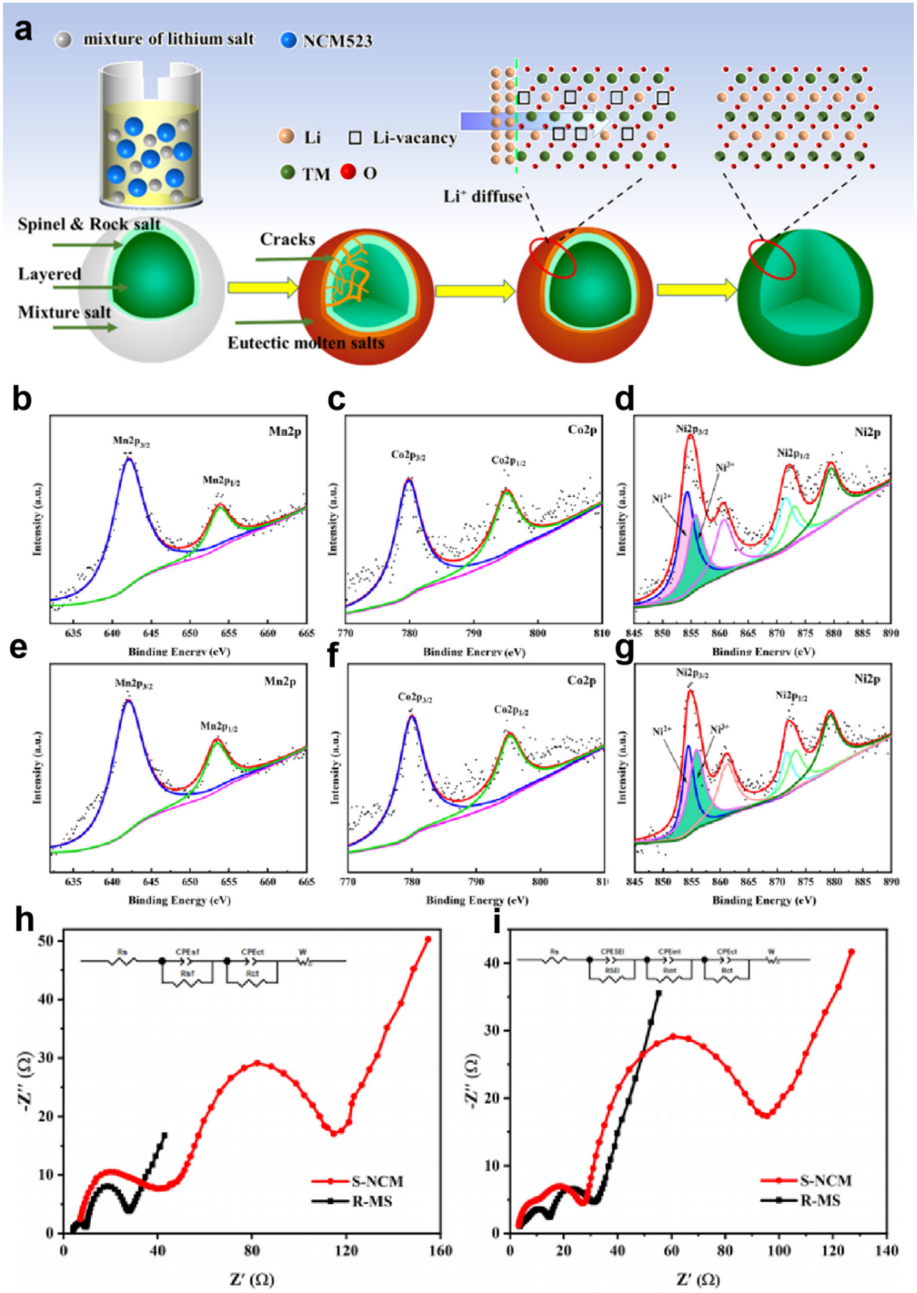
Direct regeneration of NCM523 with LiOH and Li_2_CO_3_. Reproduced with permission.^[^ ^77^ ^]^ Copyright 2020, American Chemical Society. a) Schematic diagram of the re‐lithiation process for Li composition recovery by eutectic molten salt approach. Reproduced with permission.^[^ ^77^ ^]^ Copyright 2020, American Chemical Society. b–d) XPS spectra for Mn 2p, Co 2p, and Ni 2p of S‐NCM. Reproduced with permission.^[^ ^77^ ^]^ Copyright 2020, American Chemical Society. e–g) XPS spectra for Mn 2p, Co 2p, and Ni 2p of R‐MS. Reproduced with permission.^[^ ^77^ ^]^ Copyright 2020, American Chemical Society. And Nyquist plots for S‐NCM and R‐MS at different cycle stages, with their corresponding equivalent circuit diagrams (insets): first cycle h) and 200th cycle g). Reproduced with permission.^[^ ^77^ ^]^ Copyright 2020, American Chemical Society.

The application of the molten salt method for the direct regeneration of NCM523 is not only well‐established but also supported by numerous successful examples, with highly satisfactory outcomes. For instance, using a binary molten salt system composed of LiOH and Li_2_CO_3_, the regenerated NCM523 material (R‐MS), derived from spent precursors (S‐NCM), exhibits a capacity retention rate of up to 89.06% after 200 cycles at 1C.^[^ ^77^ ^]^ The results indicate that the treatment with eutectic molten salt effectively replenishes the Li vacancies in S‐NCM and repairs its microcracks (**Figure** **13**a). During the regeneration process, the valence stability of the three elements (Ni, Co, and Mn) was ensured. XPS spectra revealed that the binding energies of Mn 2p were ≈642.2 eV (Mn 2p^3/2^) and 654.2 eV (Mn 2p^1/2^), indicating that Mn was in the +4‐oxidation state. The binding energy positions of Mn in the regenerated R‐MS material were essentially the same as those in S‐NCM, suggesting that the regeneration process did not alter the valence state of Mn (Figure 13b,e). Similarly, the binding energies of Co 2p were ≈779.0 eV (Co 2p^3/2^) and 794.1 eV (Co 2p^1/2^), confirming that Co was in the +3‐oxidation state. The binding energy positions of Co in the regenerated R‐MS material were also consistent with those in S‐NCM, indicating that the regeneration process maintained the valence state of Co (Figure 13c,f). For Ni 2p, the binding energies were ≈854.2 eV (Ni 2p^3/2^) and 873.3 eV (Ni 2p^1/2^). In the S‐NCM material, the Ni 2p^3/2^ binding energy peak was split into two peaks: 854.6 eV (Ni^2+^) and 855.7 eV (Ni^3+^), with a higher ratio of Ni^2^⁺ to Ni^3+^. In contrast, the regenerated R‐MS material exhibited an increased content of Ni^3+^ and a decreased Ni^2+^/Ni^3+^ ratio. This change is beneficial for reducing the degree of Li/Ni cation mixing and improving the electrochemical performance of the material (Figure 13d,g). Electrochemical impedance spectroscopy (EIS) analysis was conducted on S‐NCM and R‐MS materials at different cycle numbers. As shown in Figure 13h, the impedance spectrum of S‐NCM comprises two small semicircles in the high‐frequency and medium‐frequency regions, as well as a straight line in the low‐frequency region. These semicircles correspond to the Li‐ion diffusion resistance in the solid electrolyte interphase (SEI) layer (RSEI), the interfacial electronic contact resistance (Rint), and the charge transfer resistance (Rct). The impedance profile of R‐MS is like that of S‐NCM but exhibits significantly lower resistance values. After 200 cycles (Figure 13i), the regenerated R‐MS material maintains low impedance values. This indicates that the material has better structural stability and enhanced capabilities for charge transfer and Li‐ion diffusion. The results demonstrate that the regeneration process not only restores the initial performance of the material but also improves its cycling stability.

Experimental evidence confirms that Li_2_CO_3_, while conventionally employed as a precursor in LIB manufacturing, demonstrates exceptional utility in direct cathode regeneration processes. However, three fundamental considerations merit thorough investigation: First, regarding solubility constraints, the potential persistence of particulate Li_2_CO_3_ residues, even within high‐temperature molten media, requires systematic characterization. Such residual particulates may establish localized insulation domains that could detrimentally influence bulk electrical conductivity.^[^ ^120^ ^]^ Second, concerning thermal stability, the decarbonation kinetics under operational conditions present non‐trivial environmental implications. The thermodynamics of carbonate decomposition necessitate comprehensive life‐cycle assessment, particularly with respect to gaseous emissions.^[^ ^121^ ^]^ Third, from a green chemistry perspective, innovative carbon management strategies should be developed. It might be proposed that carbon capture,^[^ ^122^ ^]^ or sequestration during the regeneration process can be considered,^[^ ^123^ ^]^ and the residual waste generated from the regeneration process may also be reused.

#### 4.2.6 Direct Regeneration of LCO with Eutectic Molten Salt Method

**Figure 14:**
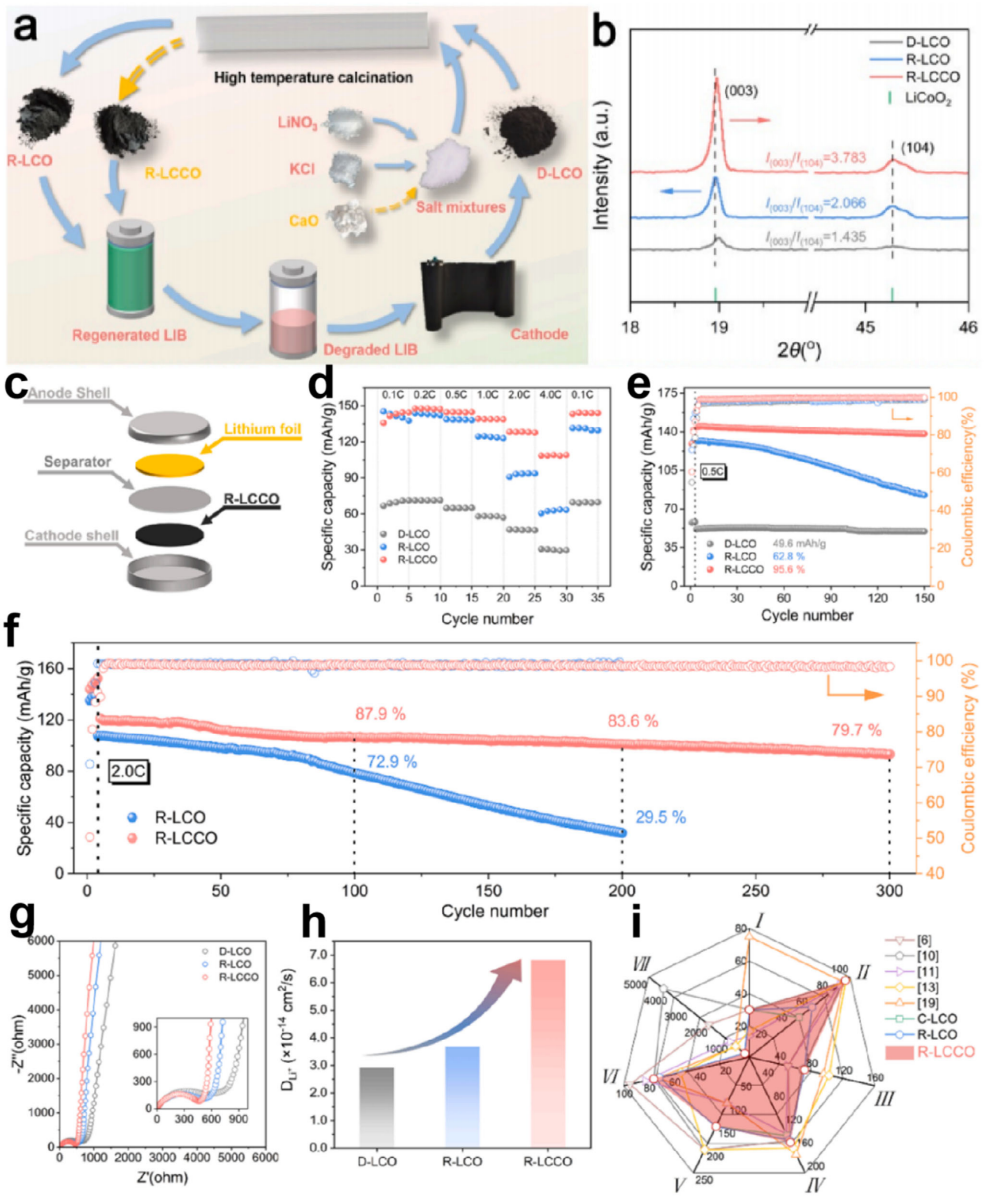
Direct regeneration of LCO with LiNO_3_ and KCl. Reproduced with permission.^[^ ^75a^ ^]^ Copyright 2024, Elsevier. a) Schematic diagram for the process of regenerating R‐LCO and R‐LCCO cathodes. Reproduced with permission.^[^ ^75a^ ^]^ Copyright 2024, Elsevier. b) XRD (003) and (104) characteristic peaks of D‐LCO, R‐LCO and R‐LCCO. Reproduced with permission.^[^ ^75a^ ^]^ Copyright 2024, Elsevier. And electrochemical performance comparison among D‐LCO, R‐LCO, and R‐LCCO: c) Schematic of the coin cell. Reproduced with permission.^[^ ^75a^ ^]^ Copyright 2024, Elsevier. d) Rate performance (1C = 150 mAh g^−1^). Reproduced with permission.^[^ ^75a^ ^]^ Copyright 2024, Elsevier. e) Cycling performance at 0.5C over 150 cycles and f) at 2.0C over 300 cycles. Reproduced with permission.^[^ ^75a^ ^]^ Copyright 2024, Elsevier. g) EIS spectra. Reproduced with permission.^[^ ^75a^ ^]^ Copyright 2024, Elsevier. h) Li⁺ diffusion coefficients (DLi⁺) calculated from EIS data. Reproduced with permission.^[^ ^75a^ ^]^ Copyright 2024, Elsevier. i) Comprehensive electrochemical performance comparison with published results: I Rate (mAh g^−1^), II Capacity retention (%), III Degraded capacity (mAh g^−1^), IV Regenerated capacity (mAh g^−1^), V Cycles, VI Replenished capacity (mAh g^−1^), VII Costs ($ kg^−1^). Reproduced with permission.^[^ ^75a^ ^]^ Copyright 2024, Elsevier.

Although there are not many examples of the direct regeneration of LCO assisted by eutectic molten salt, existing studies have demonstrated the satisfactory effectiveness of the molten salt method in regenerating degraded LCO. For instance, LCO materials regenerated directly using a eutectic molten salt system composed of LiNO_3_ and KCl exhibited a capacity retention rate of 95.6% after 150 cycles at 0.5C.^[^ ^75a^ ^]^ In this molten salt system, calcium oxide (CaO) serves as an auxiliary additive, yielding more satisfactory outcomes for direct regeneration (**Figure** **14**a). The XRD patterns (Figure 13b) reveal that the degraded LCO (D‐LCO) sample exhibits discernible (003) and (104) peaks, yet with low intensities, indicative of its inferior crystalline structure. The regenerated LCO without CaO addition (R‐LCO) shows sharper (003) and (104) peaks compared to D‐LCO, signifying partial recovery of the crystalline structure. The regenerated LCO with the addition of CaO (R‐LCCO), however, displays the sharpest (003) and (104) peaks with the highest intensities, demonstrating that the CaO‐assisted molten salt treatment leads to comprehensive structural repair and a notable enhancement in crystalline quality. The regenerated LCO materials were fabricated into coin cells for electrochemical performance evaluation (Figure 14c). The results indicate that the R‐LCCO sample exhibits remarkable rate capability at high current densities, achieving capacities of 128.1 mAh g^−1^ at 2.0C and 108.7 mAh g^−1^ at 4.0C (Figure 14d). Moreover, after 150 cycles at 0.5C, the capacity retention rate of R‐LCCO reaches 95.6%, significantly surpassing that of R‐LCO (62.8%) and commercial new LCO (C‐LCO) (50.0%) (Figure 14e). When subjected to higher rates and increased cycle numbers, R‐LCCO maintains 79.7% of its initial capacity after 300 cycles at 2.0C, whereas R‐LCO only retains 29.5% after 200 cycles (Figure 14f). This long‐term cycling performance at high current densities further corroborates the superior structural stability and electrochemical performance of R‐LCCO. In the EIS analysis (Figure 14g), the R‐LCCO sample exhibits the smallest charge transfer resistance (Rct) of 523.8 Ω, indicative of a stable electrolyte/electrode interface conducive to Li⁺ transport. Additionally, the study quantifies the Li⁺ diffusion capability, with R‐LCCO presenting a Li⁺ diffusion coefficient (DLi⁺) of 6.82 × 10^−14^ cm^2^ s^−1^, nearly double that of R‐LCO (3.67 × 10^−14^ cm^2^ s^−1^), highlighting the faster Li⁺ diffusion kinetics of R‐LCCO (Figure 14h). Ultimately, Figure 14i presents a comprehensive performance comparison of R‐LCCO in terms of rate capability, capacity retention, degraded capacity, regenerated capacity, cycle numbers, replenished capacity, and cost. The balanced and superior performance profile of R‐LCCO underscores its potential as a practical and effective regeneration strategy for spent LCO batteries.

The study demonstrates that the employed molten salt system, despite containing K^+^ and exogenously added Ca^2+^ species throughout the regeneration process, yields LCO materials exhibiting electrochemical performance comparable to or even surpassing commercial LCO products. This finding substantiates the remarkable flexibility and tolerance of the eutectic molten salt composition, where efficient Li replenishment is achieved despite the coexistence of multiple cationic species. More significantly, the eutectic molten salt system unexpectedly unlocks the high‐voltage capability of LCO. The regenerated LCO maintains structural stability even at an elevated cutoff voltage of 4.6 V, which may establish a precedent for developing LCO materials with extended high‐voltage operational limits. This discovery provides a reference case for ultrahigh‐voltage cathode design.

From a comprehensive perspective, eutectic molten salt demonstrates considerable universality in the direct regeneration of cathodes, exhibiting effective restorative effects on diverse cathode materials. Notably, multiple successful cases and varied molten salt combinations have been reported for the direct regeneration of layered NCM materials. However, it is worth emphasizing that alkaline LiOH has been frequently employed in numerous studies, likely due to its intrinsic melting characteristics, procurement cost, and regeneration performance. Nevertheless, alkaline materials may induce corrosion on reaction vessels such as crucibles, potentially increasing replacement frequency and consequently elevating process costs. Therefore, a system that balances performance, cost, and material protection warrants further exploration.

Additionally, some studies have incorporated sodium or potassium salts, which do not provide Li sources, presumably due to cost considerations and eutectic point modulation. This raises an intriguing question: could these ostensibly non‐essential cations play unique roles in the regeneration process? For instance, might Na^+^ intercalate into NCM interlayers to form stabilizing pillars, thereby enhancing structural stability?^[^ ^124^ ^]^ Alternatively, could K^+^, with its larger ionic radius relative to Li^+[^ ^125^ ^]^, occupy more space within the molten salt, thereby weakening electrostatic interactions between adjacent Li^+^ and facilitating Li^+^ diffusion?^[^ ^125^, ^126^ ^]^ These hypotheses merit systematic investigation and may provide precise insights for optimizing molten salt‐based regeneration strategies in future studies.

### 4.3 Improvements or Innovations of Eutectic Molten Salt Method for Direct Regeneration of LIBs

#### 4.3.1 Adjustment of Temperature

Rationally controlling the temperature during the regeneration process can not only repair degraded cathode materials but also reduce energy consumption.

**Figure 15:**
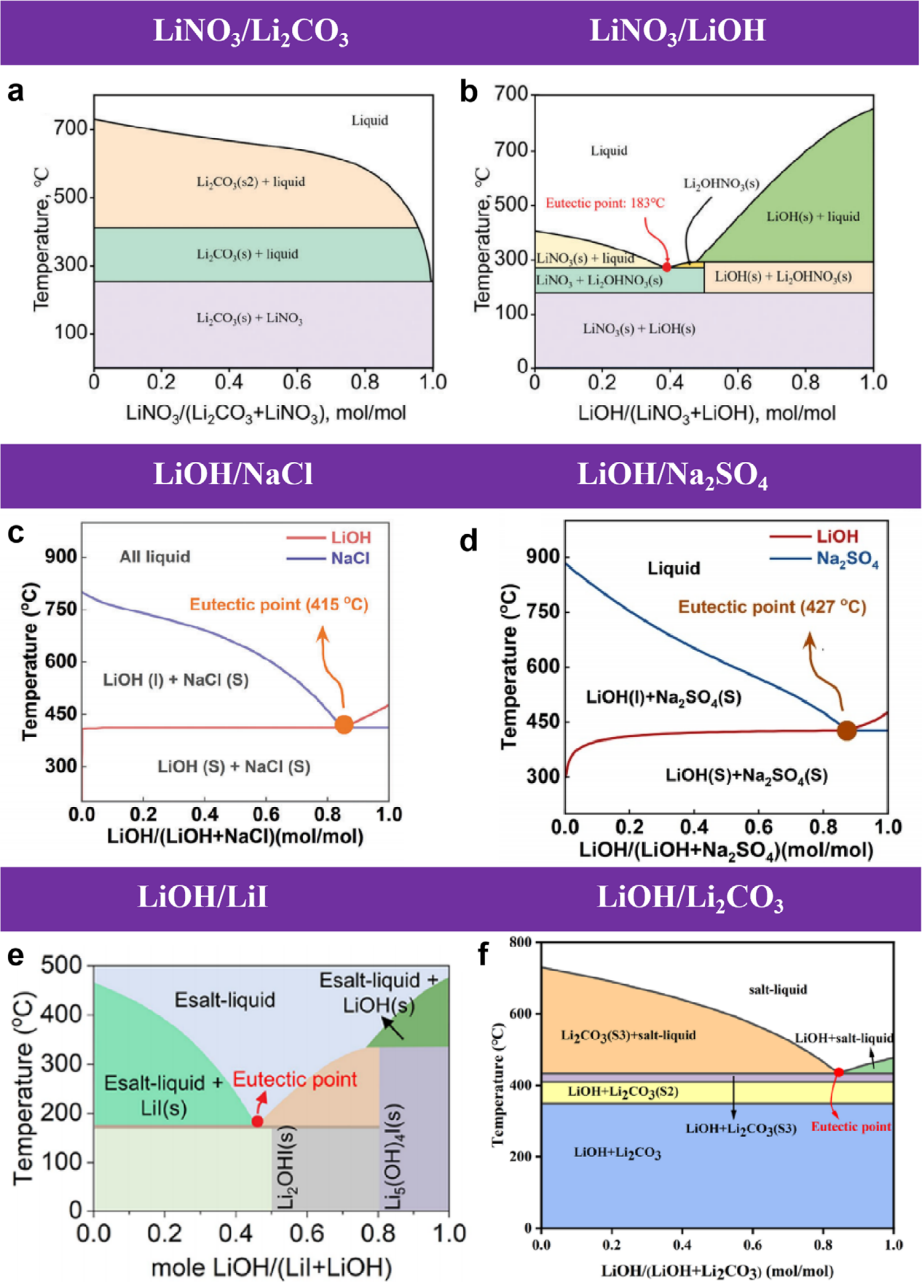
Phase diagrams of various eutectic mixtures. a) LiNO_3_/Li_2_CO_3_ mixture. Reproduced with permission.^[^ ^108^ ^]^ Copyright 2024, Wiley‐VCH. b) LiNO_3_/LiOH mixture. Reproduced with permission.^[^ ^108^ ^]^ Copyright 2024, Wiley‐VCH. c) LiOH/NaCl mixture. Reproduced with permission.^[^ ^104^ ^]^ Copyright 2023, Wiley‐VCH. d) LiOH/Na_2_SO_4_ mixture. Reproduced with permission.^[^ ^103^ ^]^ Copyright 2023, Wiley‐VCH. e) LiOH/LiI mixture. Reproduced with permission.^[^ ^71^ ^]^ Copyright 2022, American Chemical Society. f) LiOH/Li_2_CO_3_ mixture Reproduced with permission.^[^ ^77^ ^]^ Copyright 2020, American Chemical Society.

Firstly, the temperature required for the re‐lithiation process is directly influenced by the chosen molten salt combination. As shown in **Figure** **15**, different molten salt combinations have varying eutectic points, which in turn affect the minimum temperature required for the re‐lithiation process. It is evident from Table 2 that the eutectic molten salt system composed of LiNO_3_ and LiOH in a molar ratio of 3:2 has been frequently used. This is likely because its eutectic point is ≈183 °C (Figure 15b), allowing for the creation of a liquid environment for Li replenishment at a relatively low temperature.^[^ ^71^ ^]^ Similarly, researchers have also employed a eutectic mixture of LiOH and LiI, whose eutectic point is also below 200 °C (Figure 15e). Secondly, the common steps in eutectic molten salt treatment include Li replenishment and annealing. Annealing through high‐temperature calcination can restore the structure of degraded cathode materials.^[^ ^70a^ ^]^ Similarly, the annealing temperature varies depending on the characteristics of the material being treated and the molten salt combination used, and it requires researchers to gradually find the optimal solution through experiments. For example, in the direct regeneration of LFP, the high‐temperature treatment is controlled at 650 °C.^[^ ^101^ ^]^ This is not only because LFP decomposes ≈700 °C, but also because higher temperatures may cause excessive spinning of LFP particles.^[^ ^101^, ^102^ ^]^ In contrast, when directly regenerating NCM811 at high temperatures, treatment at 850 °C yields the most ideal regeneration results. However, when the temperature rises to 900 °C, the I(003)/I(104) ratio actually decreases.^[^ ^104^ ^]^ This is due to excessive temperature causing Li loss and reduced crystallinity. Therefore, optimizing the temperature for high‐temperature treatment is also very important. Finally, molten salt combinations with higher eutectic points seem to be more popular in one‐step molten salt methods. For instance, LiOH and NaCl for NCM811 (Figure 15c), LiOH and Na_2_SO_4_ (Figure 15d) are used for the direct regeneration of NCA, and LiOH and Li_2_SO_4_ for NCM622, all employing a one‐step approach with heat treatment temperatures of 750 °C or above. Although different materials require different treatment temperatures, all have achieved remarkable results.^[^ ^76^, ^103^, ^104^ ^]^

The existence of phase diagrams provides a theoretical basis for selecting either the one‐step or two‐step approach in regenerating spent cathodes using eutectic molten salt. High‐eutectic‐point systems (e.g., LiOH/NaCl) are suitable for direct high‐temperature regeneration, whereas low‐eutectic‐point systems may be more appropriate for a stepwise Li replenishment followed by annealing. However, both pathways still require optimization. The one‐step method offers operational simplicity but carries risks of Li loss due to prolonged high‐temperature exposure, along with higher energy consumption. Conversely, the two‐step process reduces energy demand but involves more intricate procedures.

Furthermore, current phase diagrams exhibit limited comprehensiveness, as certain molten salt combinations demonstrate notably depressed eutectic points. This observation prompts further exploration into predicting additional low‐eutectic‐point systems to minimize energy consumption. Computational chemistry and high‐throughput screening represent promising strategies for such predictions.^[^ ^127^ ^]^

#### 4.3.2 Auxiliary Additives

Traditional direct regeneration methods may not meet the increasingly demanding requirements for regeneration. However, the incorporation of certain auxiliary substances has enabled direct regeneration to achieve higher standards. The regenerated cathode materials have demonstrated significantly enhanced electrochemical performance.

**Figure 16:**
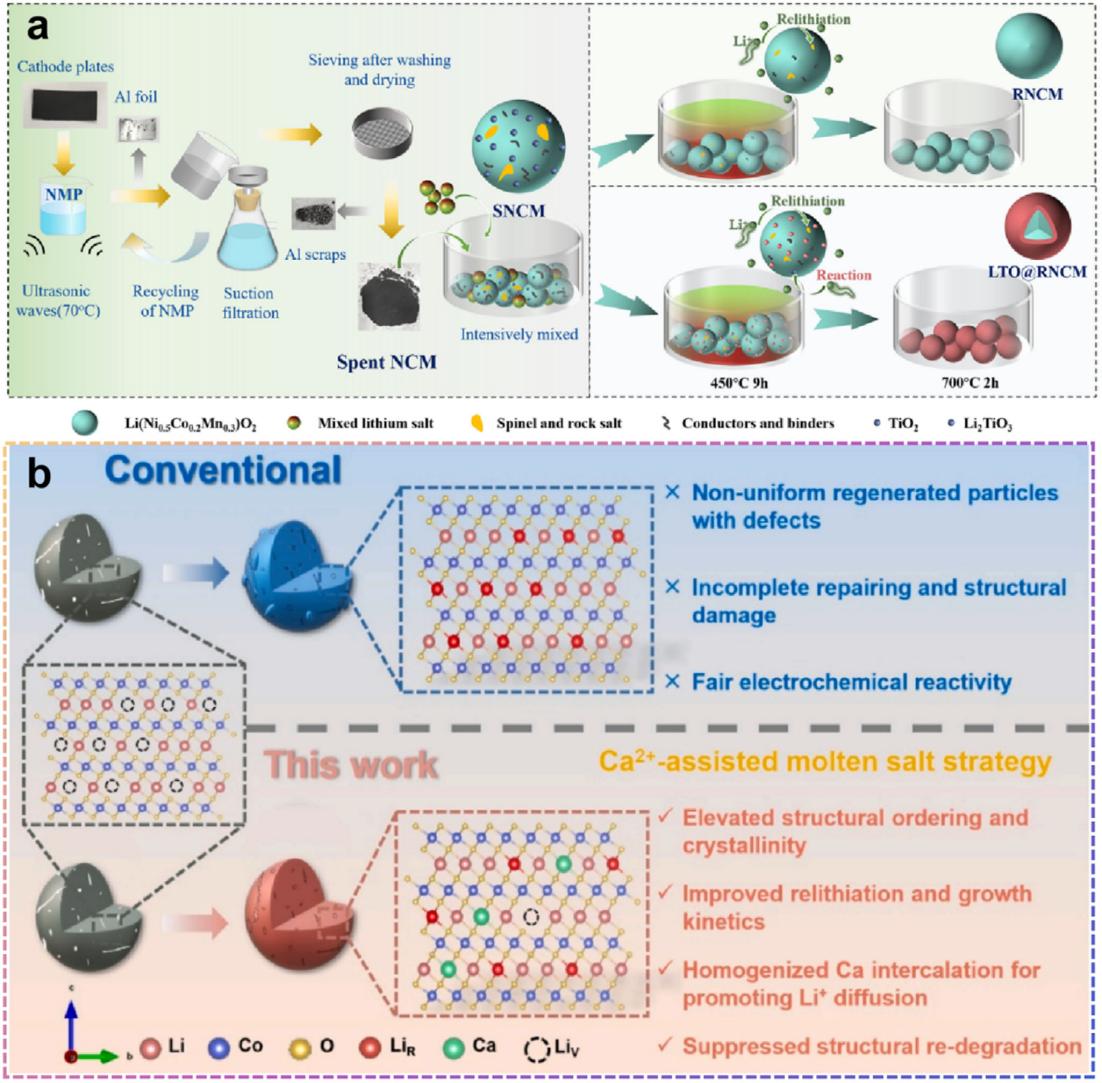
Schematic diagram of a) the regeneration process of spent NCM with TiO_2_. Reproduced with permission.^[^ ^106^ ^]^ Copyright 2020, American Chemical Society. b) the regeneration process of spent LCO with CaO Reproduced with permission.^[^ ^75a^ ^]^ Copyright 2024, Elsevier.

In the regeneration process of spent ternary cathode materials (such as NCM523), the incorporation of titanium dioxide (TiO_2_) enables the simultaneous achievement of structural recovery and surface coating formation (**Figure** **16**a). The reaction of TiO_2_ with Li salts in the molten salt generates a Li_2_TiO_3_ coating. This coating not only provides an additional protective layer for the cathode material, inhibiting erosion by the electrolyte and the dissolution of transition metal ions but also significantly enhances the diffusion rate of Li ions through its 3D Li⁺ transport channels, thereby improving the rate capability and cycling stability of the material. Moreover, this method is operationally simple and cost‐effective and can be completed at relatively low temperatures, avoiding the use of toxic compounds. It thus offers significant environmental benefits. The regenerated cathode materials exhibit electrochemical performance comparable to that of commercial materials, with a reversible capacity of 150.6 mAh g^−1^ at 1C and a capacity retention of 92% after 100 cycles. This provides a highly promising solution for the efficient recycling and reuse of spent Li‐ion battery cathode materials.^[^ ^106^ ^]^

Similarly, the degraded LCO exhibits superior electrochemical performance compared to commercial new batteries when regenerated in eutectic molten salt with the assistance of CaO (Figure 16b). The addition of CaO significantly enhances the regeneration kinetics of LiCoO_2_ in the molten salt. By modulating the growth kinetics of LCO and the Li⁺ replenishment process, CaO enables complete microstructural repair and elevated crystallinity during the regeneration process. Moreover, Ca^2+^ is homogeneously intercalated into the Li vacancies of LiCoO_2_. This intercalation not only maintains the structural stability of the regenerated material and prevents its re‐degradation during charge/discharge cycles but also promotes Li⁺ diffusion through the formation of rapid wave‐shaped diffusion pathways. Owing to these structural modifications, the regenerated LiCoO_2_ demonstrates outstanding electrochemical performance. At a high rate of 4C, the regenerated LiCoO_2_ achieves a high specific capacity of 108.7 mAh g^−1^, representing an 188.7% recovery of the performance of commercial new batteries. The incorporation of CaO involves simply adding a small amount of CaO to the molten salt, without the need for complex process updates. This regeneration method is operationally simple and cost‐effective. It avoids the use of expensive ionic liquids and toxic compounds and significantly reduces energy consumption during the regeneration process.

It is evident that auxiliary additives significantly enhance the regeneration efficacy of eutectic molten salt, demonstrating the system's high compatibility with foreign components. Notably, TiO_2_ reacts with the molten salt to in situ form a Li_2_TiO_3_ coating, eliminating the need for additional coating steps—a process both simple and cost‐effective. Meanwhile, CaO promotes Li⁺ diffusion within the molten salt through Ca^2+^ intercalation, creating a wavy Li⁺ diffusion pathway that improves rate capability.

These findings offer valuable insights for future research: exploring other oxides capable of in situ reactions to form protective coatings and releasing ions to occupy Li⁺ vacancies, thereby facilitating Li⁺ diffusion for multifunctional synergistic regeneration. Additionally, the potential catalytic activity of such oxides under high‐temperature conditions warrants further investigation.^[^ ^128^ ^]^

#### 4.3.3 Ternary Eutectic Molten Salt System

Another approach to enhance the performance of eutectic molten salt method is to upgrade binary eutectic molten salt to ternary eutectic molten salt. By incorporating a third component, the ternary eutectic molten salt system can significantly reduce the melting point, thereby achieving a molten state at lower temperatures. Owing to its complex composition, it typically exhibits a higher ionic diffusion rate. This implies that Li ions migrate more swiftly within the molten salt, enabling more efficient replenishment of the missing Li ions in the cathode material. Additionally, it can provide a richer chemical reaction environment, which is conducive to more thorough impurity removal and structural repair of the cathode material.^[^ ^75^, ^76^, ^107^, ^110^ ^]^

**Figure 17:**
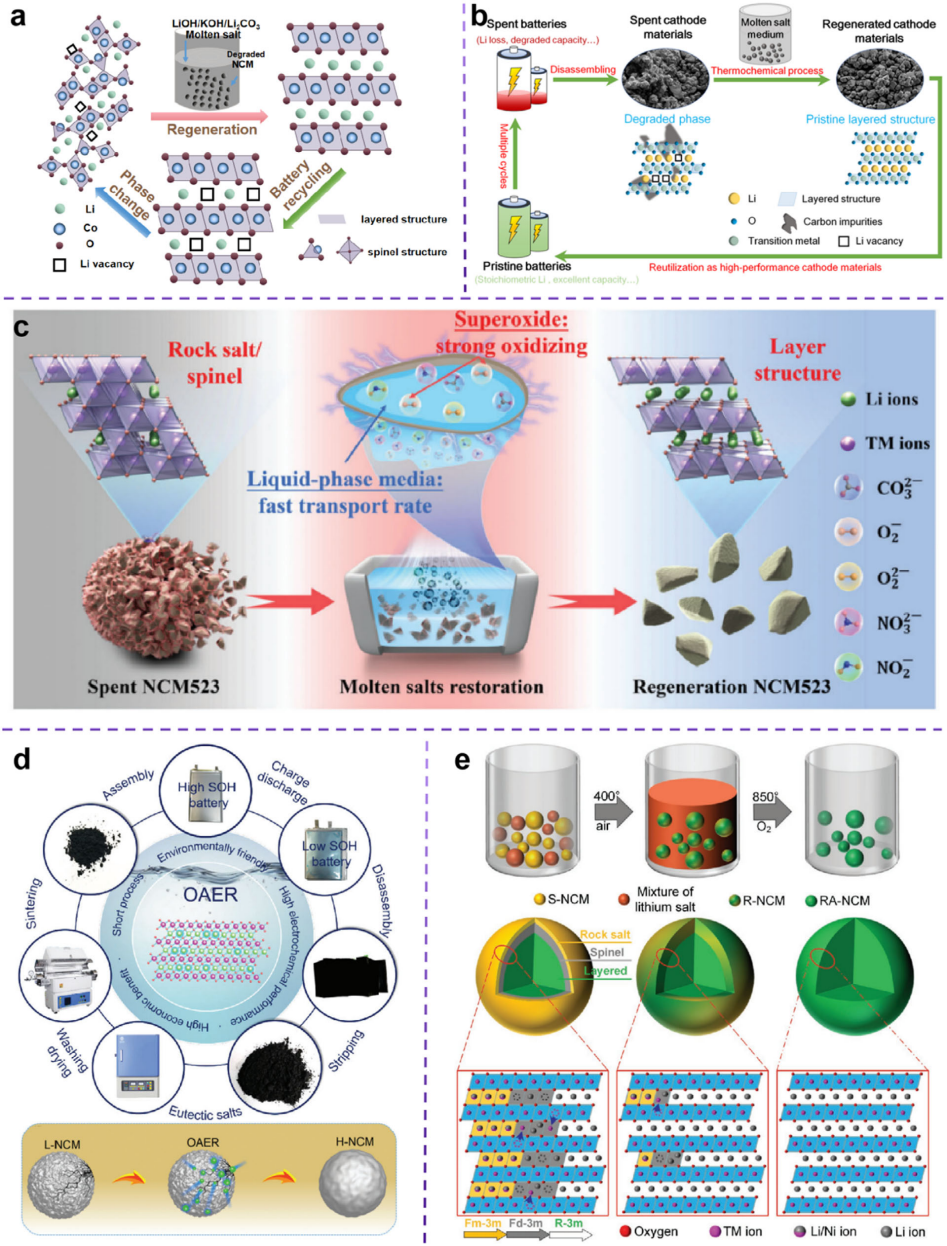
Schematic diagram for a) direct regeneration of LCO with LiOH/KOH/Li_2_CO_3,_^[^ ^110^ ^]^ and direct regeneration of NCM523 with b) KCl/KNO_3_/LiNO_3_. Reproduced with permission.^[^ ^107^ ^]^ Copyright 2020, American Chemical Society. c) LiOH/Li_2_CO_3_/LiNO_3_. Reproduced with permission.^[^ ^108^ ^]^ Copyright 2024, Wiley‐VCH. d) LiOH/LiNO_3_/LSA. Reproduced with permission.^[^ ^75b^ ^]^ Copyright 2023, Wiley‐VCH. e) LiOH/LiNO_3_/CH_3_COOLi. Reproduced with permission.^[^ ^76a^ ^]^ Copyright 2022, Wiley‐VCH.

For instance, the ternary eutectic molten salt system (LiOH/KOH/Li_2_CO_3_) demonstrates significant advantages and functions in the regeneration of degraded LCO cathode materials (**Figure** **17**a). This eutectic molten salt system possesses a high dissolving capacity, which can effectively decompose impurities within the cathode material, such as carbon and organic binders, thereby providing a pure environment for the subsequent regeneration process. Meanwhile, the ternary molten salt system offers an abundant source of Li ions, which can effectively replenish the missing Li ions in the degraded cathode material. Experimental results indicate that after treatment with the eutectic molten salt, the Li content in the degraded LCO material increased from 0.8571 to 0.977, close to the theoretical value of 1. The regenerated LCO achieved a reversible capacity of 144.5 mAh g^−1^ at 0.2C and maintained a capacity retention rate of 92.5% after 200 cycles.^[^ ^110^ ^]^

The ternary eutectic molten salt system also proves highly effective in the direct regeneration of NCM523. Characterized by a low melting point, the ternary molten salt system (KCl/KNO_3_/LiNO_3_) can achieve a molten state at relatively lower temperatures during the regeneration of degraded NCM523, thereby reducing energy consumption (Figure 17b). Meanwhile, its high ionic diffusion rate can accelerate the replenishment of Li ions, enhancing regeneration efficiency. For instance, in experiments where regeneration was conducted at 750 °C, this method was more energy‐efficient compared to traditional high‐temperature approaches and effectively facilitated the diffusion and replenishment of Li ions in the degraded cathode material. Moreover, the eutectic molten salt system possesses strong oxidizing and dissolving capabilities, which can effectively remove carbon impurities (such as acetylene black) and organic binders from the cathode material. In the experiment, after treatment with the eutectic molten salt, the carbon content in the cathode material decreased from 3.36 to 0.02 wt.%, almost completely eliminating carbon impurities.^[^ ^107^ ^]^

Notably, the ternary eutectic molten salt system (LiOH/Li_2_CO_3_/LiNO_3_) significantly facilitates the phase transformation from the rock‐salt and spinel phases to the layered structure during the regeneration of degraded NCM523 by generating highly oxidative superoxide ions (O_2_⁻) (Figure 17c). This highly oxidative environment not only accelerates the phase transformation process but also enhances its completeness. Experimental results indicate that after treatment with the ternary eutectic molten salt, the rock‐salt and spinel phases in the degraded NCM523 cathode material are completely converted to the layered structure, restoring the material's initial structure. The ternary eutectic molten salt system is not only applicable to the regeneration of NCM523 cathode materials but also exhibits broad versatility. This regeneration method can be extended to other cathode materials, such as those used in Na‐ion and Al‐ion batteries, further demonstrating the potential of the ternary molten salt system in the field of energy storage material regeneration.^[^ ^108^ ^]^

With the continuous development of eutectic molten salt, organic Li salts have also begun to be incorporated into molten salt systems for the direct regeneration of cathode materials. For instance, the addition of LSA makes great sense in the direct regeneration of NCM523 (Figure 17d). Upon reaction at high temperatures, LSA generates highly oxidative organic compounds (such as nitrobenzene and its derivatives), which promote the oxidation of Ni from lower to higher valence states, reduce the mixing of Li and Ni, and help restore the layered structure of the cathode material. The addition of LSA forms cationic and oxygen vacancies in the degraded cathode material. These vacancies facilitate the enrichment and replenishment of Li ions, thereby promoting the Li replenishment process. Theoretical calculations have shown that the incorporation of organic Li salts significantly reduces the adsorption and intercalation energies of Li in the cathode material, making it easier for Li ions to enter the lattice of the cathode material and thus promoting the Li replenishment process.^[^ ^75b^ ^]^ Also notably, the ternary eutectic molten salt (LiOH/LiNO_3_/CH_3_COOLi) can form a liquid reaction environment at lower temperatures (Figure 17e). This not only reduces energy consumption but also enhances the reaction rate and uniformity. During the reaction process, lithium acetate (CH_3_COOLi) decomposes to generate Li ions and carbon dioxide, which further facilitate Li replenishment. Moreover, the presence of organic Li salts can remove organic impurities from the materials.^[^ ^76a^ ^]^

In summary, the development of ternary eutectic molten salt represents a comprehensive advancement in the regeneration of spent cathodes via eutectic molten salt processing. By carefully designing their composition, ternary systems achieve lower operating temperatures and higher efficiency, overcoming the limitations of binary eutectic molten salt. The introduction of a third component further enhances multifunctionality—for instance, the incorporation of organic Li salts enables simultaneous oxidation, Li replenishment, and impurity removal within the eutectic system. Moreover, ternary molten salt exhibits broad material compatibility, demonstrating effective regeneration for both NCM and LCO cathodes.

Looking ahead, quaternary molten salt or ternary systems combined with auxiliary additives may soon emerge,^[^ ^129^ ^]^ offering even superior regeneration performance and expanded functionalities. However, the inclusion of additional components inevitably increases costs and raises concerns regarding material waste. Therefore, when scaling up these novel systems for industrial recycling, precise dosage optimization will be essential to balance performance and economic viability.

## 5 Technical and Economic Assessment

### 5.1 Comparison of Performance and Economy of Cathode Materials Based on Various Direct Regeneration Methods

Literatures about the direct regeneration of cathode material in the past five years has been collected, and the performance, economic benefits, and cost of the regenerative battery from its text and supporting information have also been investigated, which were compiled into **Table** **3**. The summarized methods not only include common direct regeneration methods such as eutectic molten salt method, hydrothermal method, electrochemical method, chemical re‐lithiation method, solvothermal strategy, and high temperature solid phase method, etc. Also mentioned is a technology involving a combination of supplementing metal ions, granulation, ion doping, innovative and combinatorial direct regeneration methods such as heat treatment and direct calcination.

**Table 3:** A comparative analysis of the performance and economic viability of various direct regeneration methods.

Cathode	Number of cycles (and C‐rate)	Capacity retention	Product value/Profit^b)^ [$]	Cost^c)^ ($)	Refs.
NCM622	Eutectic molten salt method				
	250 (1C)	99.4%	N.A.	N.A.	[76b]
NCM811	Solvothermal strategy				
	84 (0.5C)	91.81%	N.A.	N.A.	[130]
NCM111	Hydrothermal method				
	60 (1C)	92%	4.85	2.07	[80]
	Electrochemical method				
	300 (1C)	87.55%	N.A.	N.A.	[131]
NCM523	Green integrated recycling technology^a)^				
	200 (0.1C)	85.3%	6.25	15.84	[63]
	Direct calcination				
	1000 (0.1C)	92.7%	1.984	2.668	[70a]
	Eutectic molten salt method				
	100 (0.5C)	95.5%	N.A.	7.12^*^	[107]
	100 (0.5C)	95.6%	10.454	N.A.	[75b]
	100 (0.5C)	89.3%	N.A.	N.A.	[71]
	100 (0.5C)	93.7%	N.A.	N.A.	[76a]
	Hydrothermal method				
	200 (0.5C)	85.58%	N.A.	N.A.	[132]
	100 (10C)	82.9%	N.A.	87.88^*^	[133]
	Hydrothermal method with topotactic transformation				
	100 (0.5C)	<90%	N.A.	140.96^*^	[68b]
	Electrochemical method				
	200 (0.2C)	84.7%	N.A.	9.02^*^	[134]
	Solvothermal strategy				
	560 (1C)	90.23%	6.55	1.96	[69a]
	High‐temperature solid‐state method				
	100 (0.5C)	94.52%	N.A.	N.A.	[135]
LFP	High‐temperature solid‐state method with CNTs^d^				
LFP	100 (0.1C) 100 (0.2C)	93.8% 96.42%	21.92	7.388	[136]
	Chemical re‐lithiation method				
	400 (5C)	88.0%	N.A.	N.A.	[137]
	1000 (5C)	84.9%	N.A.	N.A.	[138]
	Hydrothermal method				
	200 (1C)	99.1%	N.A.	N.A.	[139]
	Eutectic molten salt method				
	100 (0.5C)	>90%	N.A.	N.A.	[101]
	Electrochemical method				
	300 (1C)	85.5%	5.528	1.755	[68a]
LCO	Hydrothermal method with Ni & Mn				
	100 (1C)	91.2%	N.A.	N.A.	[140]
	High‐temperature solid‐state method				
	25 (0.1C) 50 (0.5C)	78.1% 80.4%	N.A.	N.A.	[141]
	Eutectic molten salt method				
	200 (0.2C)	92.5%	N.A.	23.48^*^	[110]
	150 (0.5C)	95.6%	17.28	N.A.	[75a]

Notes: ^a)^ The technology involves a combination of supplementing metal ions, granulation, ion doping, and heat treatment; ^b)^ The profit of regenerating one kilogram of cathode material; ^c)^ The cost of regenerating one kilogram of cathode material. d: Carbon nanotubes.

Cost^*^: In the text and the supporting information (SI), there is no direct mention of prices; thus, we calculated and normalized the costs based on the prices listed on the Sigma‐Aldrich official website and the amounts used in the experimental section of the paper, with the price reference date being September 24, 2024 (LiOH: $1174.64/kg; Li_2_CO_3_: $1131.09/kg; LiNO3: $2230.94/kg; KCl: $214.13/kg; KNO_3_: $353.47/kg; KOH: $120.50/kg). N.A.: Not applicable.

Variations in recycling techniques, including raw materials and experimental conditions, lead to differing performance outcomes in recycled batteries. Notably, the direct regeneration of NCM523 batteries through the eutectic molten salt method has demonstrated promising results, with capacity retention rates exceeding 90% after 100 cycles at 0.5C, peaking at 95.6%, and dipping to a minimum of 89.3%.^[^ ^71^, ^75^, ^76^ ^]^ An outstanding regeneration with eutectic molten salt approach and the enhanced high‐temperature solid‐phase method have also reported impressive capacity retention rates of up to 95.5% and 94.52%,^[^ ^107^, ^135^ ^]^ respectively. More striking, however, is the performance of the eutectic molten salt method in regenerating NCM622. Using a low‐melting‐point eutectic mixture for one‐step regeneration, the material retained as much as 99.4% of its initial capacity after 250 cycles at 1C.^[^ ^76b^ ^]^ It is encouraging to observe the continuous improvement of these methods, achieving capacity retention rates above 80% over multiple cycles. Yet, in comparison, hydrothermal and electrochemical direct regeneration methods may slightly underperform. For instance, Zuo et al. utilized the hydrothermal method for NCM523 regeneration, yielding a capacity retention rate of 82.9% after 100 cycles at 10C.^[^ ^133^ ^]^ Similarly, Jiao et al. used the hydrothermal method, and the obtained battery had a capacity retention rate of 85.58% after 200 cycles at 1C.^[^ ^132^ ^]^ The electrochemical method's results appear less promising, with a capacity retention rate of 84.7% after 200 cycles at 0.2C.^[^ ^134^ ^]^ The emerging direct calcination method may garner widespread attention, as direct regeneration of batteries via this approach results in a remarkable capacity retention rate of 92.7% after 1000 cycles at a 0.1C charging rate.^[^ ^70a^ ^]^ It should be noted that certain methods may not yield optimal results for all NCM material types. For example, hydrothermal re‐lithiation of NCM111 material results in a capacity retention of 92% after 60 cycles at a 1C rate.^[^ ^80^ ^]^ In the case of direct regeneration of NCM811 material, the solvothermal strategy performs commendably, achieving a capacity retention of 91.81% after 560 cycles at a 1C rate.^[^ ^69a^ ^]^

As technology progresses and performance metrics advance, the focus increasingly turns to the economic feasibility of recycling processes. Though data is currently limited, there is a drive toward cost efficiency in battery recycling methods. Among these methods, the recently introduced direct calcination technique has been shown to control the direct regeneration costs of NCM523 to a mere $2.668 per kilogram, positioning it as a more economical option compared to the green integrated recycling technology, which stands at $15.84 per kilogram.^[^ ^63^, ^70^ ^]^ In terms of production efficiency, the eutectic molten salt method leads in the direct regeneration of NCM523, yielding a product value as high as $10.454 per kilogram. This significantly outperforms the direct calcination method's $1.984 per kilogram and the hydrothermal re‐lithiation method's $4.85 per kilogram for NCM111.^[^ ^70^, ^75^, ^80^ ^]^ On the other hand, certain hydrothermal methods relying solely on LiOH for Li replenishment prove to be less economical, with costs reaching a peak of $140.96 per kilogram.^[^ ^68b^ ^]^ In another study on direct regeneration via hydrothermal means, the cost of using Li carbonate also amounts to a substantial $87.88 per kilogram.^[^ ^133^ ^]^ In 2020, when the eutectic molten salt method was not yet very mature, Deng and colleagues managed to control the cost to $7.12 per kilogram when they used this method for the direct regeneration of NCM523.^[^ ^107^ ^]^ Surprisingly and excitingly, the solvothermal strategy also performs well overall, and may potentially complement and compete with the hydrothermal method in the future. When Zhou used the solvothermal strategy for the direct regeneration of NCM523, not only was a high battery capacity retention achieved, but the product profit also reached $6.55 per kilogram, with the cost being only $1.96 per kilogram.^[^ ^69a^ ^]^

The investigations reveal that the eutectic molten salt method effectively preserves the great capacity retention of regenerated materials while concurrently optimizing product profitability, it is optimistic that the eutectic molten salt method holds a promising future for the direct regeneration of NCM. Indeed, the solvothermal strategy might also emerge as a competitive and efficient method within the battery recycling sector.

Similarly, the direct regeneration technology for LFP also appears to be relatively mature, with slight improvements made based on the original process. It is not difficult to discern from Table 3 that the capacity retention of new batteries processed by various direct regeneration methods can generally reach over 85% after multiple charge‐discharge cycles. Among them, the hydrothermal method seems to have a high compatibility with LFP regeneration. Chen et al. controlled the temperature and Li^+^ concentration during the hydrothermal process, which allowed the regenerated battery to maintain a capacity retention rate as high as 99.1% after 100 cycles at 0.5C.^[^ ^139^ ^]^ The high‐temperature solid‐state method also performed well under the addition of CNTs, with the capacity retention rates reaching 93.8% and 96.42% after 100 cycles at 0.1C and 0.2C, respectively.^[^ ^136^ ^]^ The eutectic molten salt method may not have been widely adopted in the direct regeneration of LFP, or demonstrated exceptionally outstanding results; however, batteries regenerated through the eutectic molten salt process can still achieve a capacity retention rate of over 90% after 100 cycles at 0.5C.^[^ ^101^ ^]^ The re‐lithiation process through chemical means also exhibits commendable performance, with the regenerated batteries maintaining a capacity retention of 84.9% after 1000 cycles at a 5C rate,^[^ ^138^ ^]^ moreover, the introduction of organic Li salts in the chemical re‐lithiation process for direct regeneration results in batteries with a capacity retention of up to 88.0% after 400 cycles at a 5C rate.^[^ ^137^ ^]^

It is noteworthy that in the research on the regeneration of LFP, the economic requirements of the processes have also been discussed. Among them, Song et al. employed a direct regeneration process guided by high‐temperature solid‐phase synthesis, which resulted in a cost of $7.388 per kilogram, though incorporating carbon nanotubes. However, the process also demonstrated significant economic benefits, with the product value reaching as high as $21.92 per kilogram.^[^ ^136^ ^]^ Zhou et al. employed an electrochemical method for the direct regeneration of LFP, yielding new batteries with considerable electrochemical performance. After 300 cycles at a 1C rate, the capacity retention reached 85.5%, and the process cost was effectively controlled at $1.755 per kilogram.^[^ ^68a^ ^]^

Direct regeneration of LCO has also been extensively studied, although there is relatively less information on its economic benefits. However, it is encouraging to note that the eutectic molten salt method has also performed admirably in the recovery process of LCO. As early as 2020, Yang et al. successfully regenerated LCO material using the eutectic molten salt method, and the new batteries exhibited a high capacity retention of 92.5% after 200 cycles at a 0.2C rate with the process cost reaching $23.48 per kilogram, which was not considered too high at the time.^[^ ^110^ ^]^ Recent studies have indicated that the eutectic molten salt method optimized with Ca^2+^ is highly favorable for the direct regeneration of LCO. Not only does the regenerated material exhibit a capacity retention rate of 95.6% after 150 cycles at 0.5C, but the product profit also reaches a substantial $17.28 per kilogram.^[^ ^75a^ **^]^**

Direct regeneration, as an emerging battery recycling technology, has rapidly diversified in its development. From an electrochemical performance perspective, the eutectic molten salt method has demonstrated superior regeneration efficacy for both NCM and LCO cathode materials. For instance, spent NCM523 regenerated via eutectic molten salt typically exhibits a capacity retention rate exceeding 90% (up to 95.6%), significantly outperforming hydrothermal (82.9–85.58%) and electrochemical (84.7%) methods. Notably, eutectic molten salt‐treated NCM622 achieves 99.4% capacity retention after 250 cycles at 1C, while spent LCO, optimized with Ca^2+^ doping, maintains 95.6% retention after 150 cycles at 0.5C, with a profit margin of $17.28 kg^−1^.

Economically, eutectic molten salt technology has matured considerably—its cost has steadily decreased from initially high levels to $7.12 kg^−1^, with potential for further reduction, underscoring its strong scalability. However, solvothermal regeneration may emerge as a dark horse, combining low cost with competitive economic returns. While it could become a formidable rival to eutectic molten salt, a hybrid approach—leveraging solvothermal pre‐lithiation at low temperatures (150 °C) to reduce Li consumption and energy demand in subsequent eutectic molten salt processing—may also be viable.^[^ ^69a^ ^]^

At present, eutectic molten salt method remains a highly promising direct regeneration technology, balancing efficiency and cost‐effectiveness. Moving forward, as environmental, economic, and energy sustainability criteria grow stricter, the evaluation of regeneration technologies will become more multidimensional and holistic. This shift, with increased weighting on these factors, is expected to drive further advancements in eutectic molten salt methodologies.

## 6 Conclusion and Prospect

The rapid growth of the electric vehicle market has sparked a significant increase in battery demand, posing substantial economic and environmental challenges that require immediate solutions. Studies on the crystal structures of cathode materials, including NCM, LFP, and LCO, have identified degradation mechanisms such as Li loss, formation of the SEI, and the spread of internal battery fractures. This understanding provides a foundation for the direct rejuvenation of cathode materials. In recent years, the eutectic molten salt method has become a promising technique for the direct regeneration of cathode materials, with rapid progress in the selection of molten salt systems, lithiation processes, and the optimization of annealing temperatures. There is a clear trend toward developing more sustainable processes that are both environmentally friendly and economically viable. Comparative analysis of electrochemical performance, cost‐effectiveness, and product efficacy suggests that the eutectic molten salt method is particularly adept at regenerating NCM cathode materials. This approach not only ensures high battery capacity retention but also promises competitive production costs and advantages. By evaluating the production costs, potential profits, and greenhouse gas emissions associated with traditional pyrometallurgical and hydrometallurgical processes, as well as direct regeneration methods, the superiority and appeal of direct regeneration techniques can be conclusively demostrated. This evaluation highlights the potential of the eutectic molten salt method to transform the recycling and reuse of cathode materials, contributing to a more sustainable battery industry.

Nevertheless, the eutectic molten salt method is not without its limitations; based on current research, several areas could be identified for potential enhancement in the future:

### 6.1 Cost Optimization

In the quest for cost optimization of molten salt, a pressing issue remains to be addressed. The burgeoning demand for electric vehicles has led to a heightened scarcity of Li resources, causing a surge in the prices of Li salts, such as Li_2_CO_3_ and LiOH, which have remained elevated in recent years.^[^ ^142^ ^]^ Beyond the exploration of alternative or enhanced Li salt systems, as previously discussed, prioritizing Li salts with superior performance characteristics that minimize waste and inherently offer cost benefits is a strategic approach. Additionally, advancements in the production processes of Li salts, aligned with the escalating demand, are anticipated to lead to a gradual reduction in their market prices.^[^ ^143^ ^]^ The recovery and reuse of Li salts post their initial application in cathode material regeneration is another promising avenue. This includes the repurposing of discarded or surplus Li salts from experimental processes. For example, electrodialysis reversal precipitation has been successfully employed to synthesize LiNO_3_ directly from existing Li salts. This method not only facilitates the large‐scale production of LiNO_3_ in an environmentally friendly and cost‐effective manner but also broadens its potential as a precursor for the synthesis of NCM.^[^ ^144^ ^]^ In a similar vein, Fei et al. have demonstrated the efficient extraction of Li from spent Li‐ion batteries using a roasting process aided by low‐cost salts. Under optimal conditions, their method achieved a remarkable Li recovery rate of up to 99.39%.^[^ ^145^ ^]^ The future looks promising for the development of innovative Li recycling techniques, which are expected to further reduce the costs associated with cathode material regeneration processes. These advancements will not only enhance the sustainability of Li‐based technologies but also contribute to the economic viability of the electric vehicle industry. Therefore, rational selection of Li salt formulations, coupled with the reuse of Li resources and the optimization and large‐scale implementation of recycling technologies, may serve as viable strategies to reduce current Li salt costs. This approach could establish an economically sustainable cycle between LIB production and recycling within an acceptable timeframe.

### 6.2 Ball Mill Assistance

In addition to the approaches, the introduction of ball milling technology could indirectly reduce the costs associated with direct recycling processes. This mechanochemical technique not only enables the fine grinding of solid particles to the nanoscale but also creates structural defects through the milling process. This approach could enhance the material's reactivity and facilitate the regeneration process, offering a potential advantage in the recycling and recovery of battery cathode materials.^[^ ^146^ ^]^ Therefore, ball milling can reduce costs by improving reaction efficiency, thereby decreasing Li salt consumption and minimizing waste. It should be noted that temperature control is also a critical factor in the molten salt regeneration of spent cathode materials. Focusing on safety, energy efficiency, and cost‐effectiveness, it is advised to explore a eutectic molten salt method that functions under more moderate heat conditions. Building on the previously mentioned use of ternary molten salt mixtures, which optimize the system's melting and solidification points by varying the salt components, the addition of ball milling technology is considered a promising path. The mechanochemical process involves the use of a high‐energy ball mill to crush specific powders within a grinding medium. The kinetic energy produced during this operation is imparted to the powder, which breaks molecular chemical bonds and reduces particle size.^[^ ^147^ ^]^ At the same time, applying mechanical stress causes the material's crystal lattice to break apart. This is accompanied by effects like crystal deformation, an increase in the concentration of defects, and a rise in the material's internal temperature.^[^ ^148^ ^]^ These structural changes are crucial for breaking chemical bonds and creating free radicals. The energy released during this process further intensifies these reactions.^[^ ^149^ ^]^ Mechanochemical transformation efficiently uses mechanical energy, transforming it into thermal, electrical, or chemical energy. This transformation diminishes the need for external heat sources, making the reaction conditions more manageable.^[^ ^150^ ^]^ Consequently, this strategy achieves the objective of temperature reduction. Therefore, future research could explore the direct regeneration of spent cathode materials through a synergistic approach combining ball milling with eutectic molten salt at reduced or even ambient temperatures. By optimizing Li salt composition and ratios, mechanical energy input may be efficiently converted into chemical and thermal energy, enabling effective cathode regeneration. This strategy has the potential to significantly mitigate the high energy consumption and carbon emissions associated with conventional high‐temperature processes.

### 6.3 Method Combination

Subsequently, reviving cathode materials calls for a comprehensive strategy due to their inherent variety. It's unrealistic to claim that one method fits all when it comes to reviving different types of cathode materials. Reaction conditions, along with the pros and cons of each method, can vary widely, indicating that a combined approach might be advantageous. This approach would involve using the best aspects of different methods while reducing their drawbacks, customized to specific situations. For instance, literature review has revealed that the eutectic molten salt technique is particularly adept at regenerating NCM materials, whereas LFP cathodes exhibit a favorable response to hydrothermal treatments. Moreover, the combination of various techniques has already been explored by several researchers, yielding promising results. In addition to the considerable promise of ball milling for direct regeneration, other recovery techniques may also be compatibly integrated with various direct regeneration approaches to achieve synergistic effects. A case in point is the successful direct regeneration of NCM523 cathode material through a comprehensive technology that includes metal ion supplementation, granulation, ion doping, and thermal treatment ^[^ ^63^ ^]^. Similarly, the synergistic application of direct calcination followed by a cost‐effective calcination process has proven equally productive for the regeneration of NCM523.^[^ ^70a^ ^]^ These findings underscore the potential for future technological integrations to flourish and offer innovative solutions in cathode material regeneration. Future regeneration strategies for layered NCM cathode materials could integrate eutectic molten salt with ionic doping to address structural degradation. Specifically, during Li replenishment via eutectic molten salt, strategic incorporation of metal ions (e.g., Na^+^, Al^3+^) may simultaneously enhance interlayer stability through lattice doping. This dual approach may potentially eliminate the need for annealing processes, thereby addressing two critical challenges in cathode regeneration: the inevitable Li loss during high‐temperature treatment, and the substantial carbon footprint associated with conventional thermal restoration methods. For LFP cathodes demonstrating excellent compatibility with both hydrothermal treatment and eutectic molten salt processing, a sequential regeneration strategy can be employed: initial hydrothermal pre‐lithiation under mild conditions followed by abbreviated molten salt treatment, effectively reducing both energy consumption and emissions. This approach offers the additional advantage of utilizing cost‐effective Li sources during the hydrothermal stage, thereby minimizing the requirement for expensive Li salts in subsequent processing while maintaining economic viability. Indeed, these technical combinations are not limited to specific cathode materials or singular approaches but rather demonstrate significant exploration potential. By strategically integrating multiple regeneration methods according to their respective advantages in energy efficiency, cost‐effectiveness, and environmental impact, further work can tailor solutions to match the distinct characteristics of various cathode materials.

### 6.4 Environmental and Economic Considerations

Ultimately, the final stage of recovering cathode materials after regeneration requires careful thought. Beyond managing greenhouse gas and toxic emissions and their collection and remediation, there's room for improving the quantitative analysis of environmental and economic consequences. To achieve this, it's recommended to use life cycle assessment (LCA) methods and integrated techno‐economic analysis (TEA) for a thorough and quantitative evaluation of both environmental and economic impacts. These tools offer a structured way to assess the full lifecycle impacts of cathode material regeneration processes. Additionally, developing new models for calculating technological costs is encouraged. Models like the BatPac model, which uses a bottom‐up approach to cost estimation, or the EverBatt model, which combines lifecycle costing with economic analysis, can be very helpful. These models not only help assess financial implications more accurately but also serve as valuable tools in benefit‐cost accounting. They can generate comprehensive recycling process metrics, including water consumption, energy usage, operational costs, potential revenue, and greenhouse gas emissions—based on user‐selected cathode materials, input feedstock characteristics, and specified raw material pricing data. By using these advanced analytical techniques and economic models, the cathode material regeneration process can be optimized for both environmental benefits and economic feasibility. This comprehensive approach ensures that the benefits of the regeneration process are maximized while minimizing any negative environmental and economic effects.

## Conflict of Interest

The authors declare no conflict of interest.
